# Design Guidelines for Two-Dimensional Transition Metal Dichalcogenide Alloys

**DOI:** 10.1021/acs.chemmater.2c01390

**Published:** 2022-11-29

**Authors:** Andrea Silva, Jiangming Cao, Tomas Polcar, Denis Kramer

**Affiliations:** † Faculty of Engineering and Physical Sciences, University of Southampton, University Road, SO17 1BJ Southampton, United Kingdom; ‡ National Centre for Advanced Tribology Study, University Road, SO17 1BJ Southampton, United Kingdom; § Faculty of Mechanical and Civil Engineering, Helmut-Schmidt-Univeristy, Holstenhofweg 85, 22043 Hamburg, Germany; ∥ Advanced Materials Group, Faculty of Electrical Engineering, Czech Technical University in Prague (CTU), Karlovo Náměstí 13, 12135 Prague, Czech Republic; ⊥ Department of Heterogeneous Catalysis, Helmholtz-Zentrum Hereon, Max-Planck-Strasse 1, 21502 Geesthacht, Germany

## Abstract

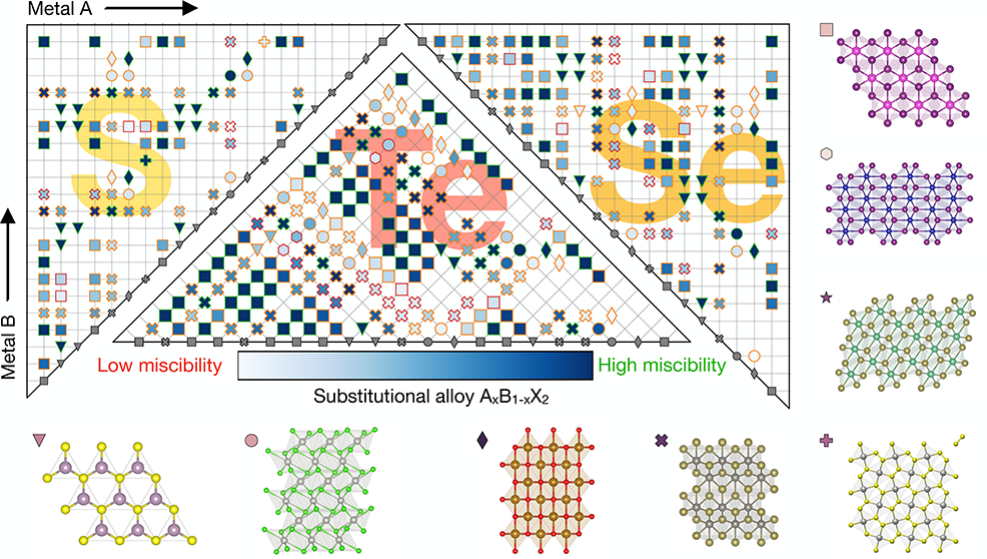

Two-dimensional (2D) materials and transition metal dichalcogenides (TMD) in particular are at the forefront of nanotechnology. To tailor their properties for engineering applications, alloying strategies—used successfully for bulk metals in the last century—need to be extended to this novel class of materials. Here we present a systematic analysis of the phase behavior of substitutional 2D alloys in the TMD family on both the metal and the chalcogenide site. The phase behavior is quantified in terms of a metastability metric and benchmarked against systematic computational screening of configurational energy landscapes from First-Principles. The resulting Pettifor maps can be used to identify broad trends across chemical spaces and as starting point for setting up rational search strategies in phase space, thus allowing for targeted computational analysis of properties on likely thermodynamically stable compounds. The results presented here also constitute a useful guideline for synthesis of binary metal 2D TMDs alloys via a range of synthesis techniques.

## I Introduction

Since the discovery of graphene, 2D materials have been a frontier in materials science and discovery. Their unique properties and reduced dimensionality have sparked an interest in nanoscale engineering applications, in addition to fundamental research interests.^1^ Ideas for 2D-material-based devices can be found in tribology,^2^ electronics,^3^ and catalysis,^4^ among other areas. Up to now, most research efforts have focused on identifying 2D unaries and binaries both theoretically^5−7^ and experimentally^8,9^ with only limited attempts to exploit the vast chemical space spanned by alloys to optimize properties. Therefore, little is known about their thermodynamic phase behavior. The structures and orderings of possible alloys are largely unexplored territory.^10,11^ A few 2D ternaries have been reported experimentally,^12,13^ but no systematical analysis across chemical spaces has been carried out, although a handful of binary alloy systems have been studied.^14−16^ But knowledge of their thermodynamic properties is fundamental for rationally advancing the engineering applications of 2D materials. For instance, the presence of miscibility gaps and competing ternaries has to be taken into account when properties such as bandgap and electronic transport are tuned to desired values by chemical doping.^17^

Due to superior scalability, computational tools can complement experimental efforts by efficiently scanning phase space to provide guidelines for synthesis and estimates of properties, potentially reducing the number of viable candidates by orders of magnitude. For example, Mounet et al.^5^ reduced a data set of 1 × 10^5^ bulk crystal structures from experimental databases to 258 easily exfoliable monolayer (ML) candidates, which has to be compared with dozens of candidates that are usually the subject of large-scale experimental studies.^8,9^

Empirical rules like the Hume–Rothery rules^18^ and bulk Pettifor maps^19^ have guided the discovery of metallic bulk alloys in the last century. The wide validity of these simple rules in metallic alloys is somewhat surprising but has been comprehensively verified by a symbiotic relationship between experiments and simulations. The phase diagrams of these bulk alloys have been mapped out experimentally since the 1940s and later integrated with and rationalized with predictive theories enabled by the advent of Density Functional Theory (DFT) and Cluster Expansion (CE) methods^20^ in the 1980s. More recently, empirical rules have been cast in terms of probabilistic models trained on computational data sets^21^ or extended to include the physics of oxides.^22^

Here, we compile a data set of two-dimensional TMD compounds in different prototypes and explore technologically relevant alloying possibilities on both the metal and chalcogenide sites. The results allow us to extend the Hume–Rothery rules to this class of materials and build Pettifor maps for substitutional alloys as a visual tool to navigate the chemical space of two-dimensional TMDs. Selected predictions by this map are benchmarked against DFT calculations and experimental results from the literature, achieving remarkable agreement.

## II Chemical and Coordination Spaces

**Figure 1:**
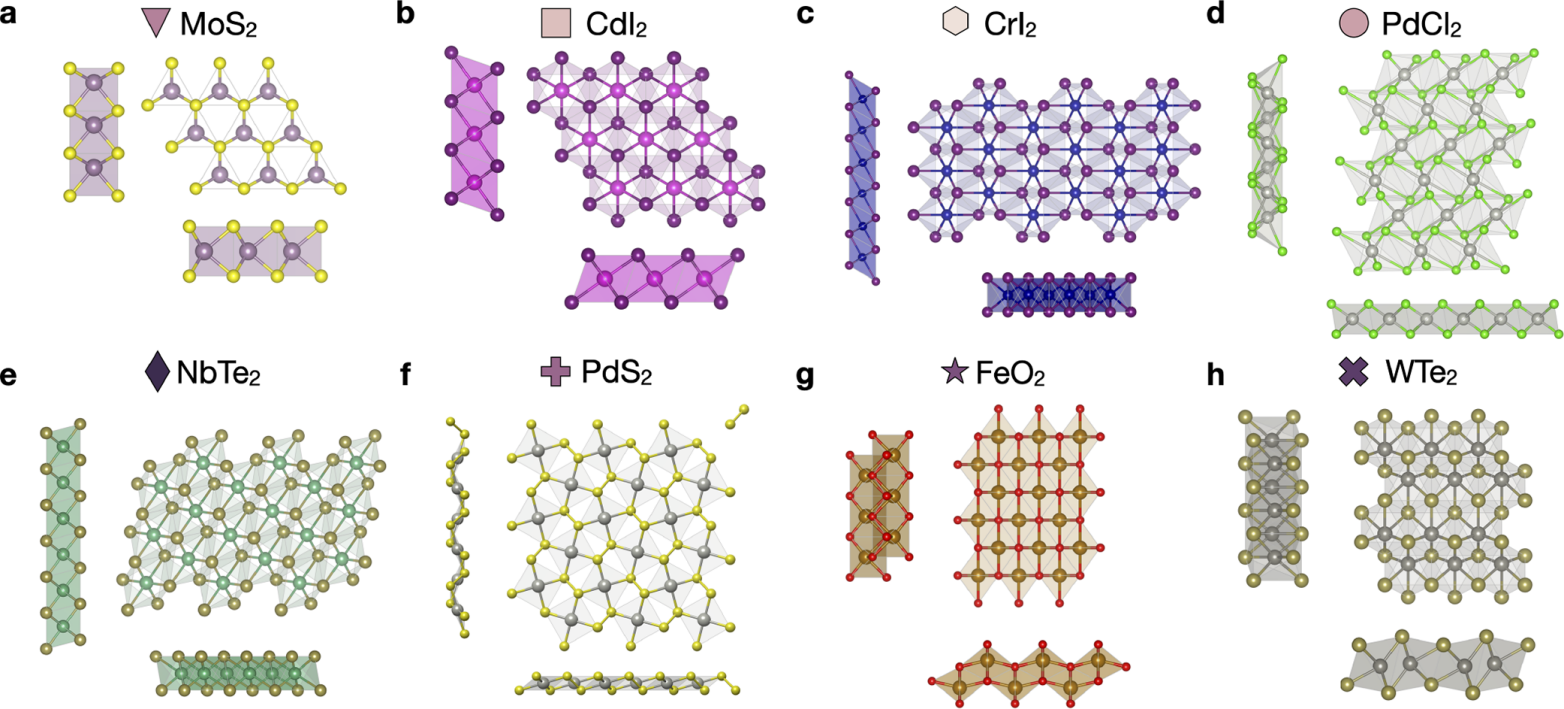
(a–h) Side and top views of the eight MX_2_ prototypes. The prototype will be identified in the rest of the paper by the symbol to the left of the name. The space group of each prototype is reported in Table SIII of the SI.

The space of considered structural prototypes for 2D TMD alloys is built from the database compiled by Mounet and co-workers,^5^ comprising 258 mechanically stable ML structures identified from experimental bulk compounds. Thus, the phase stability study is conducted on ML geometries only. The selection of the possible prototypes and elements to mix is further guided by literature knowledge^5,9,23,24^ to filter the original database according to the class of materials of interest. The database is scanned for compounds of the form MX_2_, where M is a TM cation and X is the anion, oxidizing the TM (see Section I in the SI for the list of cations and anions considered). While selecting the prototypes, the possible cations are restricted to the transition metals considered, but the anions are not limited to chalcogenides, because layered prototypes that could host TMD alloys may not be expressed in terms of chalcogenides in the database (see Section I and Table SII in the SI for details). This search yields the *N*_*p*_ = 8 prototypes shown in Figure 1a–h, whose space groups are reported in Table SIII of the SI.

Intermediate TMs (Cr, Mn, Fe, Ru, Os) are considered here, although they do not form layered chalcogenides on their own but might form ML alloys in combination with other TMs, e.g., Fe-doped MoS_2_ ML.^23^ Late transition metals from group XI onward are excluded, as they do not bind with chalcogenides to form layered materials.^9^ This yields *N*_M_ = 21 TMs as possible cations M in the MX_2_ stochiometry, with X = (S, Se, Te) as possible *N*_X_ = 3 anions.

While the methodology described here is valid for any stochiometry and cation–anion selection, our analysis will focus on MX_2_ compounds, as these are the most frequently synthesized and studied compounds of the family. This selection yields *N*_M_ × *N*_X_ × *N*_*p*_ = 504 binaries as a starting point for *N*_M_^2^ × *N*_X_ × *N*_*p*_ = 10584 substitutional alloys on the TM site and *N*_X_^2^ × *N*_M_ × *N*_*p*_ = 1512 substitutional alloys on the chalcogenide site. The total number of candidates, although large from an experimental point of view, allows for an exhaustive theoretical analysis rather than approximate methods based on statistical sampling of configurational space.^25^

The energy above the ground state of each compound MX_2_ in a given prototype *p*, also known as *lattice stability*, is given by the total energy per site with respect to the ground state (GS).^26,27^ For varying TM and fixed chalcogenide, the lattice stability reads
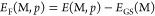
1where *E*(M, *p*) is the minimum energy of the compound in prototype *p* per number of sites *n* in the metal sublattice, i.e., the number of TMs in the unit cell. The offset energy for each TMD, *E*_GS_(M), is the minimum energy across the prototype space *E*_GS_(M) = min_*p*_*E*(M, *p*). As the starting points are ML geometries, the present analysis is especially relevant for experimental techniques able to bias the synthesis toward atomically thin films.^28^ At the same time, the results presented here for a monolayer could, in principle, be extrapolated to bulk layered TMDs, as the binding energy between the layers (typically around 10 meV/atom for TMDs^29,30^) does not usually affect the single layer phase behavior.^31^

Finally, the lattice stability definition in eq 1 is easily adapted to fixed metal M and varying chalcogenide X, i.e., *E*_F_(X, *p*) normalized to the number of sites in the chalcogenide sublattice.

**Figure 2:**
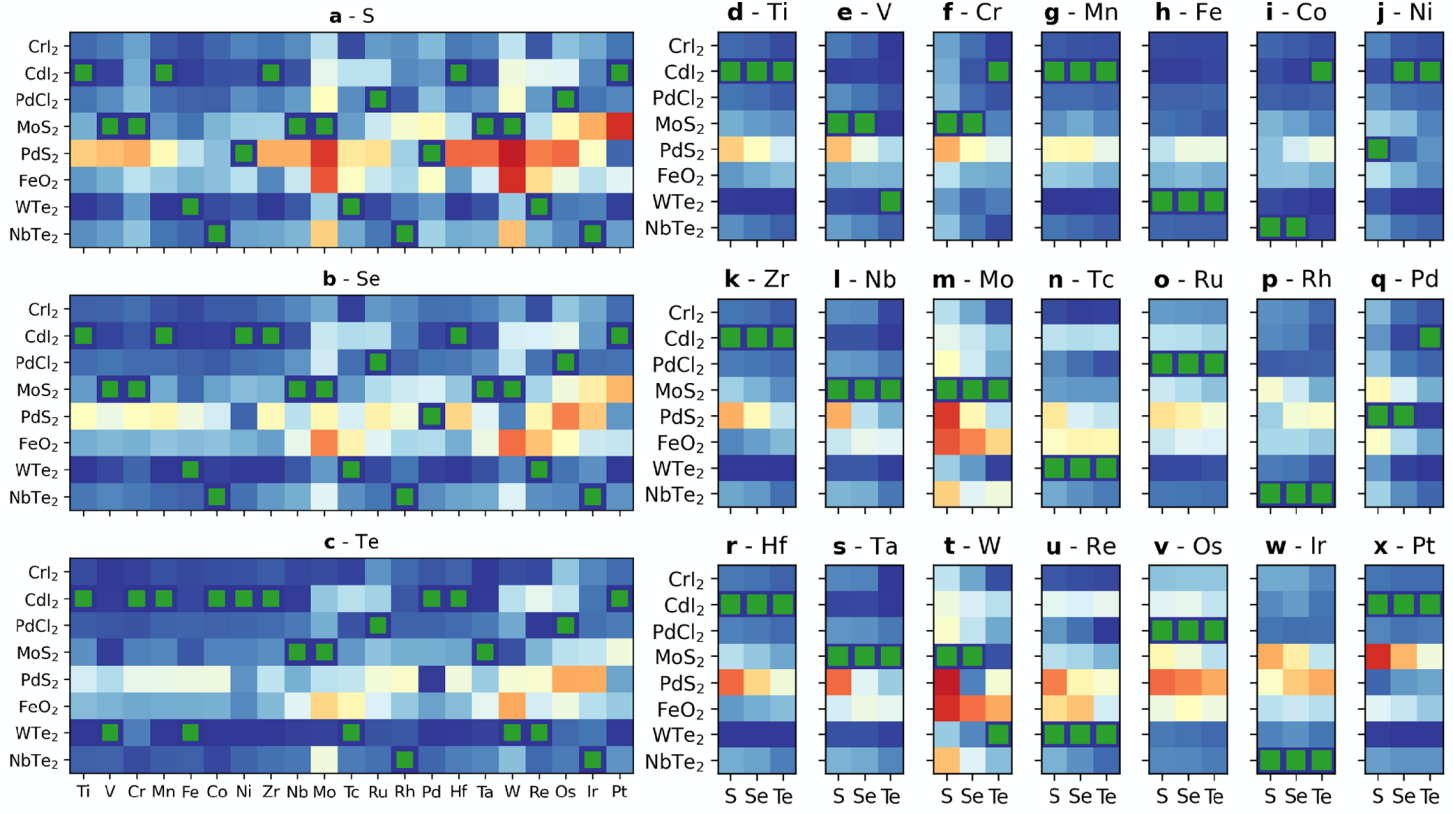
Lattice stability of MX_2_ compounds for (a) X = S, (b) X = Se, and (c) X = Te and (d–x) for fixed chalcogenide and fixed TM. The prototypes (shown in Figure 1a–h) are indicated on the *y* axis. The varying element (M or X) is indicated on the *x* axis. The color scale reports the energy above the ground state in eV per lattice sites from deep blue (*E*_F_ = 0 eV/site) to bright red (*E*_F_ = 2 eV/site). Green squares mark GS within the eight prototypes, defined by *E*_F_ = 0 in the corresponding column. The numerical value associated with each entry in panels (a–c) and (d–x) are reported in the SI Table SIV and Table SV, respectively.

Figure 2a–c reports the energy above the ground state per lattice site defined in eq 1 for the selected TMs at fixed chalcogenide and Figure 2d–x for the selected chalcogenides at fixed TM. Each column shows the energy above the ground state of the given TMD MX_2_ in the eight prototypes with respect to the identified 2D GS (green squares). Blue shades designate low energy prototypes, while yellow to red shades designated high energy prototypes.

Known coordination trends in layered compounds are identified correctly. TMDs based on *d*^2^-metals (Ti, Zr, and Hf) favor the octahedral coordination of the p-CdI_2_ prototype (Figure 1b) for all chalcogenides.^32^ The GS of transition metal sulfides based on *d*^4^ metals (Cr, Mo, W) is the prismatic prototype p-MoS_2_ (Figure 1a), while the GS coordination switches to octahedral for CrTe_2_ and WTe_2_ (see Figure 2t).^32,33^

An important chemical trend emerges by comparing the lattice stabilities in the different chalcogenide spaces in Figure 2a–c: the average energy above the GS reduces from the sulfides to the tellurides. This trend can be understood in terms of the evolution of the bond character between the metal and chalcogenide: the bonds in tellurides are more covalent than in sulfides. The charge redistribution in these strongly covalent bonds can change the GS prototype, e.g., by enhanced metal–metal bonding^34^ or by reduced energy penalties of non-GS coordination environments.

Figure 2d–x reports the lattice stability for fixed metal and varying chalcogenides in all considered prototypes. Trends for varying anion X are simple compared with the varying metal case: in most cases, the same ground state is found for S-, Se-, and Te-based TMDs, and the GS prototype follows the metal period, e.g., all *d*^2^ metals (Ti, Zr, Hf) favor p-CdI_2_ for any chalcogenide. The origin of this regular behavior can be explained in terms of coordination chemistry: the prototype stability is mostly dictated by the *d* manifold of the metal. This has implications for alloying on the chalcogenide site. In the majority of cases, where the GS geometry is the same for two chalcogenides, alloying on the X site at a fixed metal should be thermodynamically favorable to tailor properties, as discussed further below. On the other hand, those rarer cases where the GS prototype changes with the chalcogenide, e.g., the W-based TMDs in Figure 2t, could harbor interesting polymorphism and phase transitions as a function of the concentration of the substituting element; this case is discussed in detail in Section V.D and compared with experimental data.

Finally, it is important to realize the scope of validity and possible sources of errors in the data set presented here. Spin-polarized DFT calculations are used. Hence, nonmagnetic and ferromagnetic GS are correctly described. Antiferromagnetic (AFM) orderings are not considered, as calculations are performed in cells comprising a single TM site. To the best of the authors knowledge, the only AFM orderings for the considered stoichiometry are reported for NiS_2_ and MnS_2_.^35^ While important for materials properties, the AFM GS in layered TMDs is usually almost degenerate in energy with FM states^35^ and represents a second order effect in phase stability that has been excluded here for the sake of manageable computational effort. Moreover, no Hubbard correction (GGA+U) is included here. The effect of Hubbard U on the relative total energy for the considered TMD stoichiometry is negligible,^35^ but a detailed benchmark must be carried out when applying our protocol to different stoichiometries, as discussed in the Methods section. Moreover, as the M–X bonds develop a more covalent character from X = S → Te, pronounced charge redistribution may occur in specific orderings of Q_1–*x*_M_*x*_Te_2_ systems, yielding a significant change in formation energy, i.e., the formation of ternary compounds. This deviation from the pristine compounds behavior cannot be captured by the metric defined below. A telluride case where the predictions of our metric are verified is discussed in Section V.D, but care must nonetheless be taken when exploring the tellurides more generally.

## III Metastability Metric in the Ideal Solid Solution

An intuitive approach to explore which metals are likely to mix in a given chalcogenide host (and vice versa) is the ideal solid solution limit, a noninteracting model based on the lattice stability of pristine, binary TMDs defined in eq 1. As for the lattice stability in eq 1, we focus first on substitution on the TM site; the generalization to the chalcogenide site is straightforward and briefly outlined afterward. Given a binary pseudoalloy on the metal site in a prototype *p*, M_*x*_Q_1–*x*_X_2_|_*p*_, the ideal solid solution represents a model with negligible interactions between the fraction *x* of sites occupied by M and the remaining 1 – *x* sites occupied by Q. In the ideal solid solution model, the behavior of a prototype *p* in energy-composition space is represented by the line connecting the energy above the ground state of QX_2_ at *x* = 0 with the energy above the ground state for MX_2_ at *x* = 1 in the same prototype, e.g., the elements (Q, *p*) and (M, *p*) of the matrix in Figure 2a–c, respectively. Hence, in the ideal solid solution model, the energy above the ground state of a mixed configuration at concentration *x* is given by
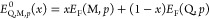
2By construction, this energy is exactly zero everywhere if M and Q share the same GS structure *p*, i.e., *E*_F_(M, *p*) = *E*_F_(Q, *p*) = 0. In any other case, the energy will be positive: if we suppose the metal M has a GS geometry *p*′ ≠ *p*, the fraction *x* of material MX_2_|_*p*_ would transform into *p*′ to reach equilibrium at zero temperature.

The model effectively quantifies the metastability at zero temperature of alloys in a selected prototype *p* as a function of concentration *x*. By construction, this model cannot predict stable mixtures, i.e., negative formation energies, but can be used to estimate the likelihood of solubility and phase separation in a system: the lower the metastability of the solid solution model, the smaller any stabilizing mechanisms must be to enable alloy formation under synthesis conditions. For example, entropy could stabilize solid solutions at finite temperature. The equilibrium of an alloy in the prototype *p* at temperature *T* is determined by the free energy *F*_Q,M,*p*_(*x*, *T*) = *E*_Q,M,*p*_^0^(*x*) – *TS*(*x*), where the configurational entropy of an ideal binary alloy is *S*(*x*) = −*k*_*b*_[*x* log *x* + (1 – *x*) log(1 – *x*)]. It weights all possible configurations of the two atom types on the metal sublattice equally and is a function of the concentration *x* only, independent of the elemental pairs.^36^ This stabilization mechanism will be discussed in detail below in relation to experimental synthesis temperatures. At zero temperature, electronic effects may likewise stabilize orderings, especially in the Te-based TMDs, where covalent bonds may lead to strong mediated interactions between metal sites.^27,34^

**Figure 3:**
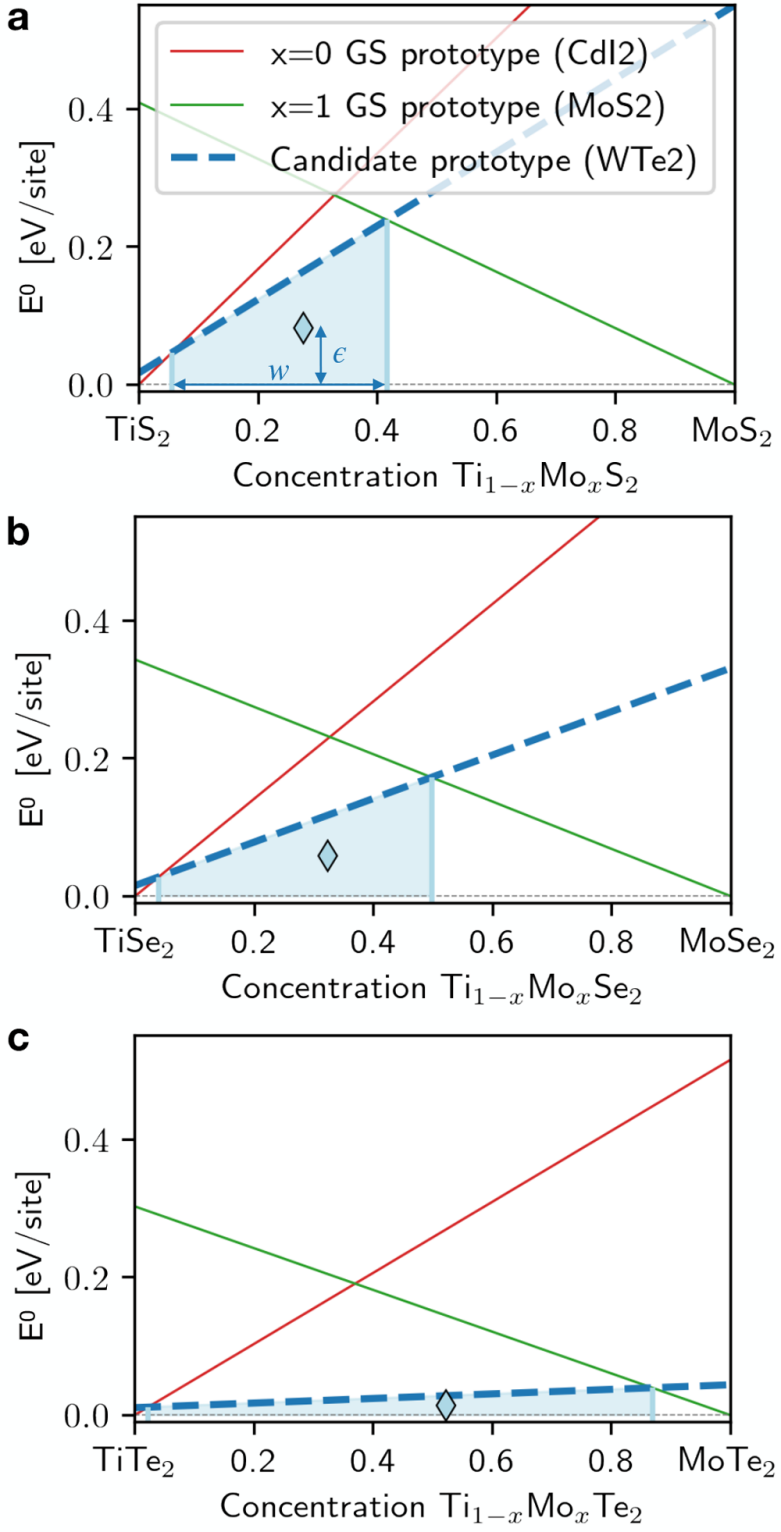
Construction of the metastability metric for TMD compounds based on Mo and Ti transition metals and different chalcogenides (a) (Ti:Mo)S_2_, (b) (Ti:Mo)Se_2_, and (c) (Ti:Mo)Te_2_. The ground state prototypes are CdI_2_ for TiX_2_ (solid red line) and MoS_2_ for MoX_2_ (green line). The candidate prototype is WTe_2_ (dashed blue line). Light blue areas highlight the extent of the metastability window in the energy above the ground state-concentration (*x*, *E*) space. Blue diamonds mark the centroids of the metastability window. The height of the centroid ϵ and the window width *w* are the arguments of the ranking function in eq 3.

A metric in the composition-energy space is used to compare the relative metastability of pseudobinary alloy candidates. We focus first on metal site substitutions and consider a prototype *p* and two chalcogenides MX_2_ and QX_2_ with GS prototype *p*_M_ and *p*_Q_, respectively. The convex hull across all phases in the concentration-energy space is the line *E* = 0 connecting the energies of the end-members in their respective GS prototypes, i.e., the dashed gray lines in Figure 3. A point on this line at the fractional concentration *x* ≠ 0, 1 represents a phase separating system where the fraction *x* of MX_2_ is in its GS prototype *p*_M_ and the remaining 1 – *x* is in its own GS *p*_Q_. For a configuration to be stable, its energy must be lower than this hull. As our model by definition cannot break this hull, we characterize the metastability of a model alloy by its positive energy above the ground state, i.e., its distance from the hull.^37^

We define a descriptor intended to capture the energetic “disadvantage” of a particular prototype (*p*, Q, M) relative to the relevant binary ground states as follows. The metastability window of the (*p*, Q, M) triplet is defined as the range of concentration *x* where the distance from the hull given by eq 2 within the prototype *p* is lower or equal to the distance from the hull within the GS prototypes *p*_M_ and *p*_Q_, as shown by blue regions in Figure 3. The metastability metric characterizes this window in term of its width *w* along the concentration axis (see light-blue vertical lines in Figure 3) and the height of the energy penalty centroid of the window (see light-blue diamond in Figure 3). The same construction applies to substitution on the chalcogenide site at fixed metal, i.e., two compounds MX_2_ and MY_2_ with GS prototypes *p*_X_ and *p*_Y_, respectively.

Let us apply this construction to an example: consider the solid solution model of the (Mo:Ti)S_2_ alloy shown in Figure 3a. The solid red line refers to the energy distance from the hull along the tie line Ti_1–*x*_Mo_*x*_S_2_ of the p-CdI_2_ prototype, which is the GS of TiS_2_ at *x* = 0, i.e.,  in eq 2. The solid green lines refers to the ground state of MoS_2_, with . The dashed blue line refers to the candidate prototype p-WTe_2_, which is the GS of neither, i.e., . The distance from the hull of these prototypes varies as a function of the concentration: the GS prototypes are favored near the respective end-members, e.g., p-MoS_2_ in the range *x* ∈ [0.4, 1] in Figure 3a. The candidate prototype p-WTe_2_ provides a lower energy metastable solution than the two end member GS prototypes in the range *x* ∈ [0.1, 0.4]: the corresponding metastability window is assigned the width *w* and the energy penalty ϵ highlighted in Figure 3a. The metastability metric is sensitive to the chemistry of the system also at a fixed cation: the metric evolves for different cations X = (S, Se, Te) as shown in Figure 3a–c.

The possible scenarios are the following: (i) When the two TMDs share the same prototype GS, the distance from the hull in that prototype is zero everywhere and the metastability window extents from *x* = 0 to *x* = 1. In this case, solubility is likely and the metastability metric is *w* = 1, ϵ = 0. (ii) When the candidate prototype *p* is the GS for one of the pristine compounds, the metastability window extends from the extremal concentration, *x* = 0 or *x* = 1, up to the intercept with the ground state of the other compound. (iii) For non-GS prototypes, there could be a metastability window of finite width 0 < *w* < 1 and energy penalty ϵ > 0, or the metastability window does not exist, when the distance from the hull of the candidate prototype is higher than either GS prototypes for any concentration. In the latter, phase separation in that prototype is likely and the metastability metric is *w* = 0, ϵ > 0.

Applying the construction depicted in Figure 3 to all TM pairs yields a *N*_M_ × *N*_M_ matrix, for each prototype *p* and each chalcogenides X. Conversely, applying the construction to all chalcogenide yields a *N*_X_ × *N*_X_ matrix, for each prototype *p* and each of the *N*_M_ = 21 metals. Each entry of these *metastability matrices* is a 2 × 2 matrix containing the bounds of the metastability window and the energy above the ground state in eq 2 evaluated at the metastability limits, i.e., minimum and maximum hull distance within the window. The matrices associated with each prototype are reported in Section III of the SI.

## IV Optimal Prototypes for Alloys

Given two TMDs, we identify the prototype most receptive for substitutional alloying on the metal or chalcogenide site by ranking the metastability metric of all TM_1_-TM_2_-prototype (or X_1_-X_2_-prototype) triplets. The following parametric function assigns a single value to the metastability windows
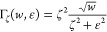
3where *w* is the width of the metastability window and the energy penalty ϵ is the hull distance of the centroid defined by the window in the energy-concentration space, i.e., blue diamonds in Figure 3. The ranking function is normalized between zero and one, Γ_ζ_(*w*, ϵ): *w* ∈ [0, 1]ϵ ∈ [0, *∞*] → [0, 1]: it associates zero to “bad” candidates and one to “good” candidates. In detail, all zero-width windows are mapped to zero, Γ_ζ_(0, ϵ) = 0 ∀ϵ, while the highest score is assigned to the combination of maximum width and null energy penalty, i.e., Γ_ζ_(1, 0) = 1. Effectively, the function encourages large metastability windows *w* and discourages large energy penalties ϵ. Details regarding the ranking function and the selection of the appropriate weight, ζ = 0.080 eV/site for the present data set, are reported in Section V of the SI.

**Figure 4:**
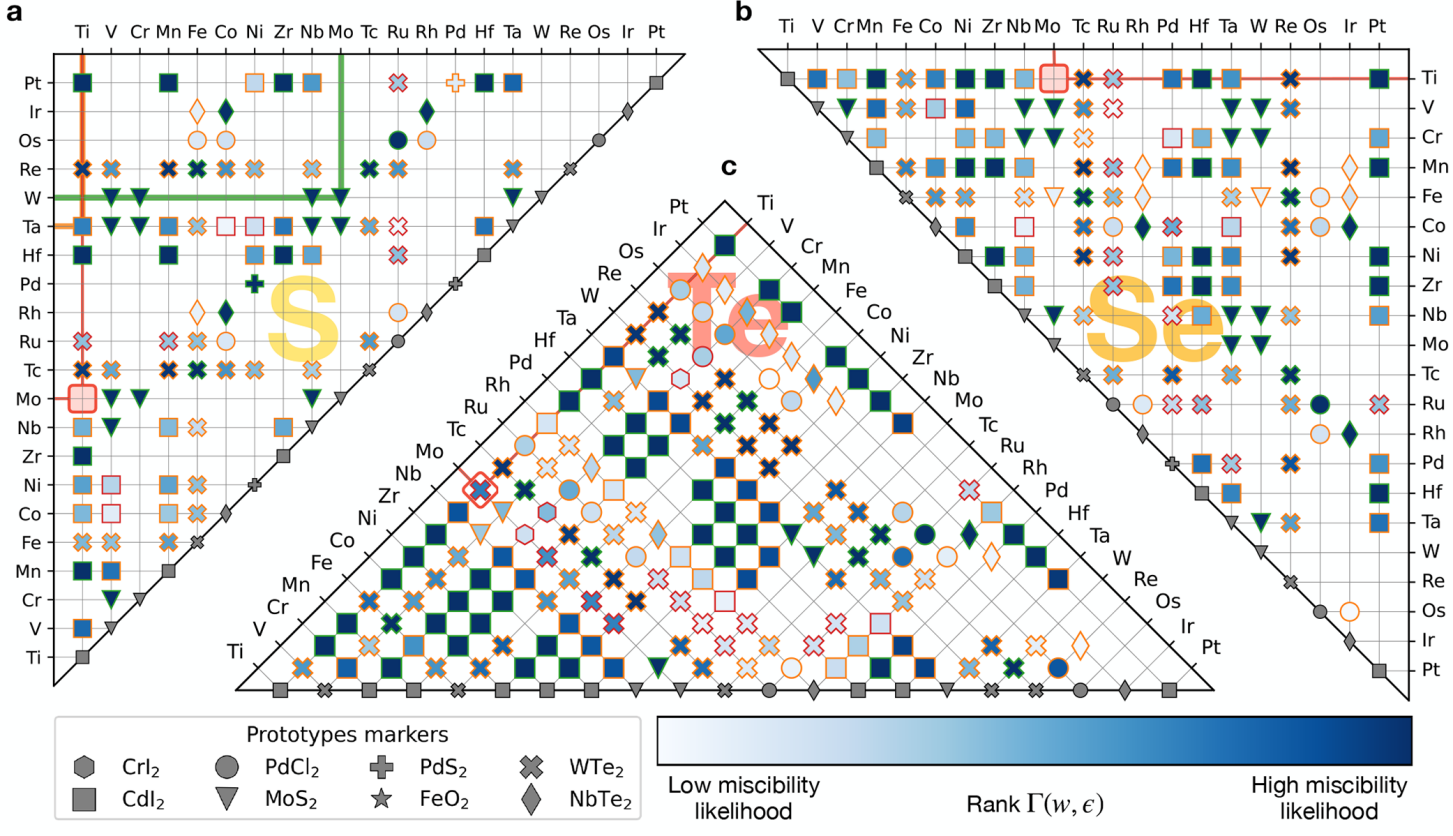
Pettifor maps for the optimal prototype for Q_*x*_M_1–*x*_X_2_ pseudobinary alloys at fixed chalcogenide (a) X = S, (b) X = Se, and (c) X = Te. The prototypes are ranked using Γ_ζ_ with ζ = 0.080 eV/site. The color code refers to the likelihood of the alloy according to the ranking in eq 3; see the colorbar on the bottom right. The edge color of each marker indicates whether the optimal prototype is the ground state of both (green), one (orange), or neither (red) of the pristine TMDs comprising the (M:Q)X_2_ mixture. Marker-prototype correspondence is reported in the legend at the bottom left. Gray markers on each diagonal of each panel a, b, and c report the GS prototype of the corresponding pristine TMD MX_2_. Green, orange, and red lines serve as a guide to the eye toward the entries corresponding to the examples discussed in the main text. For a version without rotation and example lines see SI Section VI, Figures S13–16.

The optimal prototypes for substitution on the metal site are shown in Figure 4 for each pair of transition metals. The color code of each entry shows the ranking of Q_1–*x*_M_*x*_X_2_ in the optimal prototype; see the marker legend. Additionally, the edge of each marker indicates whether that prototype is the ground state of both (green edge), one (orange edge), or neither (red edge) pristine compounds. Figure 4 provides a visual tool to navigate the possible mixtures of transition metals within the chalcogenide planes. Large blue marks in Figure 4 indicate a high rank (small energy penalty and wide metastable window) and, thus, that miscibility between the two metals within the chalcogenide host is likely. On the other hand, white marks indicate a low score (high energy penalty and small metastable window) that likely results in miscibility gaps.

The distinction between likely mixing and likely separating systems can be further constrained by extending the Hume–Rothery rules to our case:^18,27^ miscibility between transition metals within a chalcogenide host is expected if the lattice mismatch between the pristine compounds is less than 15%^18^ (see SI Section IV for the definition and values of the mismatch in these compounds) and the energy above the ground state of the optimal prototypes is below a threshold of *E* = 120 meV/site, as metastable compounds within this range have been observed experimentally.^37^ As a result, Figure 4 features “missing elements” where the optimal prototypes are unlikely to be receptive to alloying due to large lattice mismatch or high energy above the ground state. A different layout of the optimal prototype maps, with the full information on the energy penalty and window size, is reported in SI Figures S16 and S17. Note that the maps become more populated going from sulfides to tellurides. This is in agreement with the lowering of the energy landscape with increasing covalency that is also seen in Figure 2. For a quantitative visualization of this trend, see Figure S18 in the SI.

As an example of how to navigate the map, consider the pseudobinary Mo_1–*x*_W_*x*_S_2_. Following the green lines in the sulfides map, Figure 4a leads to a deep blue triangle with green edges, indicating the maximum ranking for p-MoS_2_, which is the GS of both compounds. This corresponds to the maximum likelihood to mix.

As another example in the sulfides, consider the Ti_1–*x*_Ta_*x*_S_2_ pseudobinary, whose entry is highlighted by orange lines in Figure 4a. The map reports as the optimal geometry p-CdI_2_, which is the GS of TiS_2_, but not of TaS_2_ (GS prototype p-MoS_2_), hence, the orange edge. The marker color is blue (but lighter than in the best-rank previous example Mo_1–*x*_W_*x*_S_2_), signaling that alloying is still likely even in the non-native host. This prediction is discussed in detail in the next section.

Finally, Mo–Ti-based TMD alloys provide an example of varying phase behavior in different chalcogenide spaces. The entries in the sulfide, selenide, and telluride cases are highlighted by red lines and squares in Figure 4a,b,c. In the S and Se spaces, the entry is missing, signaling that the metals are likely to phase separate according to the generalized Hume–Rothery rules. But, the likelihood of forming an alloy increases in the tellurides, as signaled by the light-blue cross (p-WTe_2_) in Figure 4c. This trend is consistent with the low lattice stability penalty in tellurides seen in Figure 2c and with the evolution of the metastability metric reported in Figure 3: the lattice stability of p-WTe_2_ on the Mo-rich side *x* = 1 reduces significantly along S → Se → Te from *E*_F_(Mo, WTe_2_) = 0.55 eV/site for S over 0.33 eV/site for Se to 0.04 eV/site for Te. Consequently, the centroid energy and metastability window width (light blue diamond and area in Figure 3) become lower and wider, respectively, yielding a favorable ranking Γ in the tellurides. The stability of the distorted octahedral structure of WTe_2_ has been attributed to an increase in direct metal–metal bonding;^34^ we speculate that the same argument could apply for MoTe_2_ in p-WTe_2_, given the chemical similarity between Mo and W. This pseudobinary alloy is further characterized in the next section.

As a first benchmark, the information in Figure 4 can be compared with alloys reported in the literature. We first focus on alloys of the most studied pristine compound in the TMD family: MoS_2_. Zhou and co-workers^8^ recently reported synthesis of (Nb:Mo)S_2_ MLs, which is shown as likely to mix in Figure 4a. However, the same work reports a (Mo:Re)S_2_ ML alloy, whose metastability window is small and high in energy (see Figure 4a and SI Figure S2). Another recent report^38^ characterizes (V:Mo)S_2_ MLs experimentally, which is also a TM pair likely to mix according to our analysis. The first example mentioned above, (Mo:W)S_2_ (green lines in Figure 4a), has also been realized in experiments.^16,39^ Finally, V-doped WSe_2_ and (Mo:W)Se_2_ alloys have been recently synthesized^40−42^ as non-MoS_2_-based examples that are both indicated as likely miscible alloys in Figure 4b.

**Figure 5:**
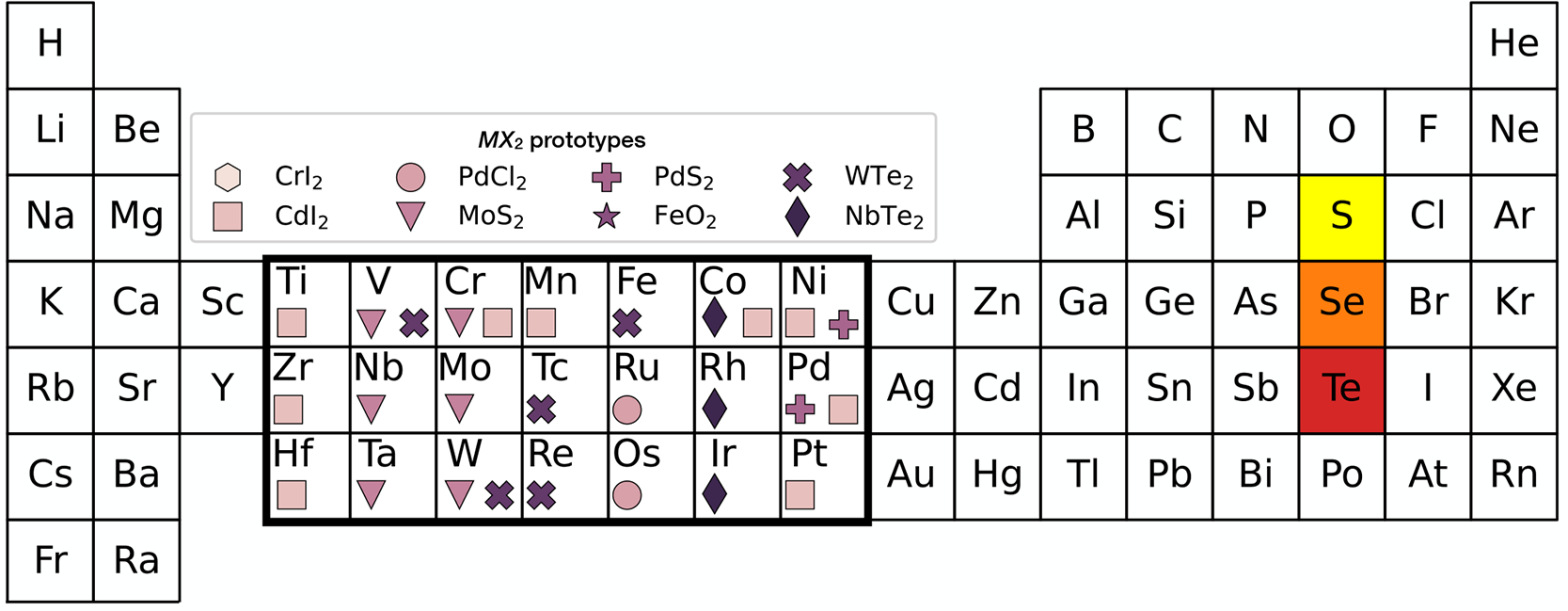
Polymorphism for  pseudobinary alloys at fixed metal *M* (entries highlighted by the thick black rectangle in the periodic table) and varying chalcogenide X, Y (colored entries in the periodic table). Marker-prototype correspondence is reported in the legend at the top center. The leftmost symbol in each metal M entry corresponds to the most receptive prototype for any substitutional alloy on the chalcogenide site, i.e., , , and . The rightmost marker (if any) corresponds to the second best prototype for chalcogenide mixtures. For the optimal prototype matrices underlying this polymorphism map see the SI Figure S17.

The optimal prototype is not very sensitive to a change of the chalcogen atom at fixed metal, as discussed in Section II. The optimal geometry for pseudobinary alloys on the chalcogenide site is, therefore, predominantly the common GS prototype. This allows us to report the most likely prototypes for a given TM across the chalcogenide spaces in Figure 5, which is a condensed version of the maps in Figure 4. The most and second-most receptive prototypes for alloys M(X_*x*_Y_1–*x*_)_2_ are given for each of the considered cations. Figure 5 is convenient to gauge likely TM coordinations for each of the transition metals.

Consider the Ti entry as an example: the GS is p-CdI_2_ for S, Se, and Te (see Figure 2d), and thus alloying on the chalcogenide site is most likely to occur in this prototype. When GS prototypes differ between chalcogenides at a fixed metal, alloy possibilities in non-native prototypes may arise. Tungsten exhibits this type of polymorphism: the dominant prototype (left symbol) is p-MoS_2_, the GS prototype of WS_2_ and WSe_2_, but a second symbol is added on the right for the homonymous prototype p-WTe_2_. Figure 5 can be benchmarked against limited experimental data. The same-prototype alloys Mo(S:Se)_2_, Mo(S:Te)_2_, and W(S:Se)_2_ have been synthesized.^42−46^ W(Se:Te)_2_ is a confirmed case of polymorphism between two prototypes. This system is analyzed in detail and compared with available experimental data^47^ in the next section.

## V Orderings in Pseudobinary Alloys

The phase behavior predicted by the Pettifor maps in Figure 4 is benchmarked by sampling the configurational space at varying concentration with electronic-structure calculations. For substitution on the metal sublattice, the formation energy of a pseudobinary alloy M_*x*_Q_1–*x*_X_2_ is obtained by taking the GS end members as reference for the ordered configuration σ(*x*) at concentration *x*:
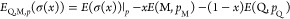
4where *E*(σ(*x*))|_*p*_ is the total energy per lattice site of the configuration σ(*x*) in the host lattice defined by the prototype *p*. *E*(M, *p*_M_) and *E*(Q, *p*_Q_) are the total energies per lattice site of MX_2_ and QX_2_ in their GS prototypes *p*_M_ and *p*_Q_, respectively. This chemical reference assures that the formation energy in eq 4 at end-member concentration *x* = 0 and *x* = 1 corresponds to the energy above the ground state reported in Figure 2.

The set of geometrically distinct orderings is generated using CASM.^48−50^ The geometries are fully relaxed, including cell shape and volume. The relaxation of the cell is needed to accommodate possible lattice mismatch. This extra degree of freedom, however, may result in non-GS prototypes transforming into more stable ones of similar symmetry.^51^ For details see the Methods section.

The following section reports a computational test of the predictions discussed in the previous section (highlighted by colored lines in Figure 4). These cases represent one strongly and one weakly phase-separating substitutional alloy on the TM site, an alloy on the TM site with finite-miscibility in a non-native prototype already at zero temperature, and a case of polymorphism for alloying on the chalcogenide site.

### V.A Strong Phase Separating: (Mo:Ti)S_2_ Pseudobinary Alloys

**Figure 6:**
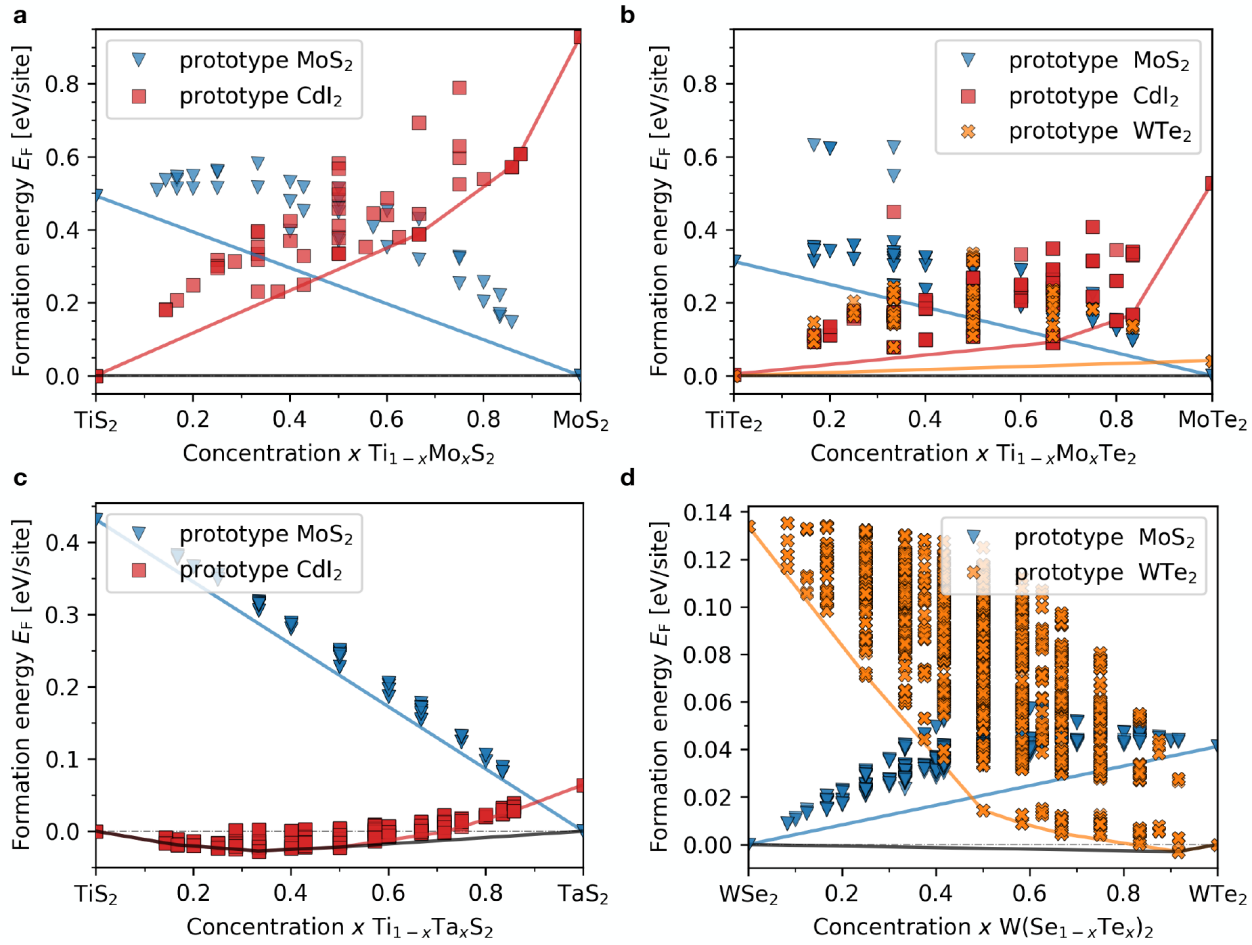
Formation energies in eV/lattice site computed from DFT calculation across the tie-line concentrations of the pseudobinary alloys: (a) (Ti:Mo)S_2_, (b) (Ti:Mo)Te_2_, (c) (Ti:Ta)S_2_, and (d) W(Se:Te)_2_. Different shapes and colors refer to different prototypes as reported in the legend. Color-matching solid lines report the convex hull construction within each host, marking the thermodynamic stability at the fixed host. The black solid line in each plot shows the interhost convex hull.

As already discussed, the high lattice stability of Mo in p-CdI_2_ and Ti in p-MoS_2_(see Figure 3a) results in a low ranking of the metastability metric; hence, the corresponding missing entry in Figure 4a (or the high-energy solutions in Figure S1 in SI). The phase separation prediction is confirmed by total energy DFT calculations of the ordered configurations as shown in Figure 6a. No configurations in the p-MoS_2_ prototype (blue symbols) display lower formation energy than the solid solution model (solid blue line). Within the p-CdI_2_ prototype, some configurations display a lower energy compared to the solid solution model; see points on the solid red line. This electronic stabilization mechanism, however, is not enough to break the interprototype convex hull (dashed-dotted gray line at *E* = 0), resulting in an overall phase separating system. The origin of this zero-temperature phase behavior lies in the different local environments favored by each TM, as explained in terms of crystal field levels in ref (31). The effect of temperature is explored in ref (31) by means of Monte Carlo simulations based on a cluster expansion Hamiltonian, trained on the DFT data set.^52^ The finite temperature phase diagram indicates that the miscibility gap in Figure 6a closes above the melting temperature of the compounds and that only a small percentage of doping near the end-members is possible due to configurational entropy, in agreement with experimental estimations.^53^

### V.B Weakly Phase Separating: (Mo:Ti)Te_2_ Pseudobinary

While the Mo and Ti phases separate within the S host, the metastability metric suggests that alloying should be possible within the Te host, in the p-WTe_2_ prototype (see Figure 4c). Figure 6b reports the benchmark of this prediction. The formation energy of all configurations is significantly lower in this case, although not enough to break the interhost hull at zero temperature (black line in Figure 6b). At the same time, this DFT search confirms the higher likelihood of alloying in this case with the miscibility gap expected to close at lower temperatures than those reported for (Mo:Ti)S_2_.^31^

Note that some configurations in the p-CdI_2_ and p-WTe_2_ prototypes overlap on the Ti-rich side of Figure 6b. The p-WTe_2_ host can transform into the GS p-CdI_2_ prototype in our computational protocol. Although symmetry is conserved during relaxation, a varying number of point group operations are removed by TM substitutions, which gives sufficient degrees of freedom to change the prototype. The similarity between the two structures driving this transition is quantified in terms of a structural descriptor similar to the SOAP kernel^54,55^ in the SI Section VII.A and Figure S20. Because of this overlap, the convex hull in Figure 6b can be expressed in terms of the p-MoS_2_ prototype and an octahedral-like prototype, comprising the structures derived from p-CdI_2_ and p-WTe_2_. The intrahost convex hull of this hybrid prototype (see purple line in SI Figure S21a) lies less than 50 meV from the interhost convex hull (black line in Figure 6b), and miscibility should occur up to *x* = 0.23 at synthesis temperatures of around 900 K, see SI Section VII.A and Figure S21b for details.

### V.C Cross-Host Miscibility: (Ti:Ta)S_2_ Pseudobinary Alloys

We now test the prediction from the ranking map in Figure 4a presented in section IV for interhost high miscibility against actual alloy configurations from DFT. Figure 6c reports the formation energy of (Ti:Ta)S_2_ alloys in the p-CdI_2_ (red symbols) and p-MoS_2_ prototypes (blue symbols). As predicted by the metastability metric, TiS_2_ and TaS_2_ segregate in p-MoS_2_: no configuration lies below the solid solution limit (straight blue line); see SI Figure S1 for the relative entry in the metastability matrix. In the p-CdI_2_ prototype, the native host for TiS_2_ but not for TaS_2_, the alloyed configurations lie below the cross-host solid-solution hull (dashed-dotted gray horizontal line) from *x* ≈ 0 up to *x* ≈ 0.7. While at zero temperature only the configurations on the interhost convex hull (black solid line) are stable, the energy scale is small compared to room temperature, suggesting that synthesis of solid-solution alloys in the p-CdI_2_ prototype is achievable experimentally, e.g., with CVD techniques. Indeed, there are reports of (Ti:Ta)S_2_ solid solution alloys in the literature,^56^ although no crystallography data or solubility limits are available to date. This experimental confirmation further validates the predictive power of our approach.

### V.D Polymorphism Chalcogenide Alloys: W(Se:Te)_2_ Pseudobinary

**Figure 7:**
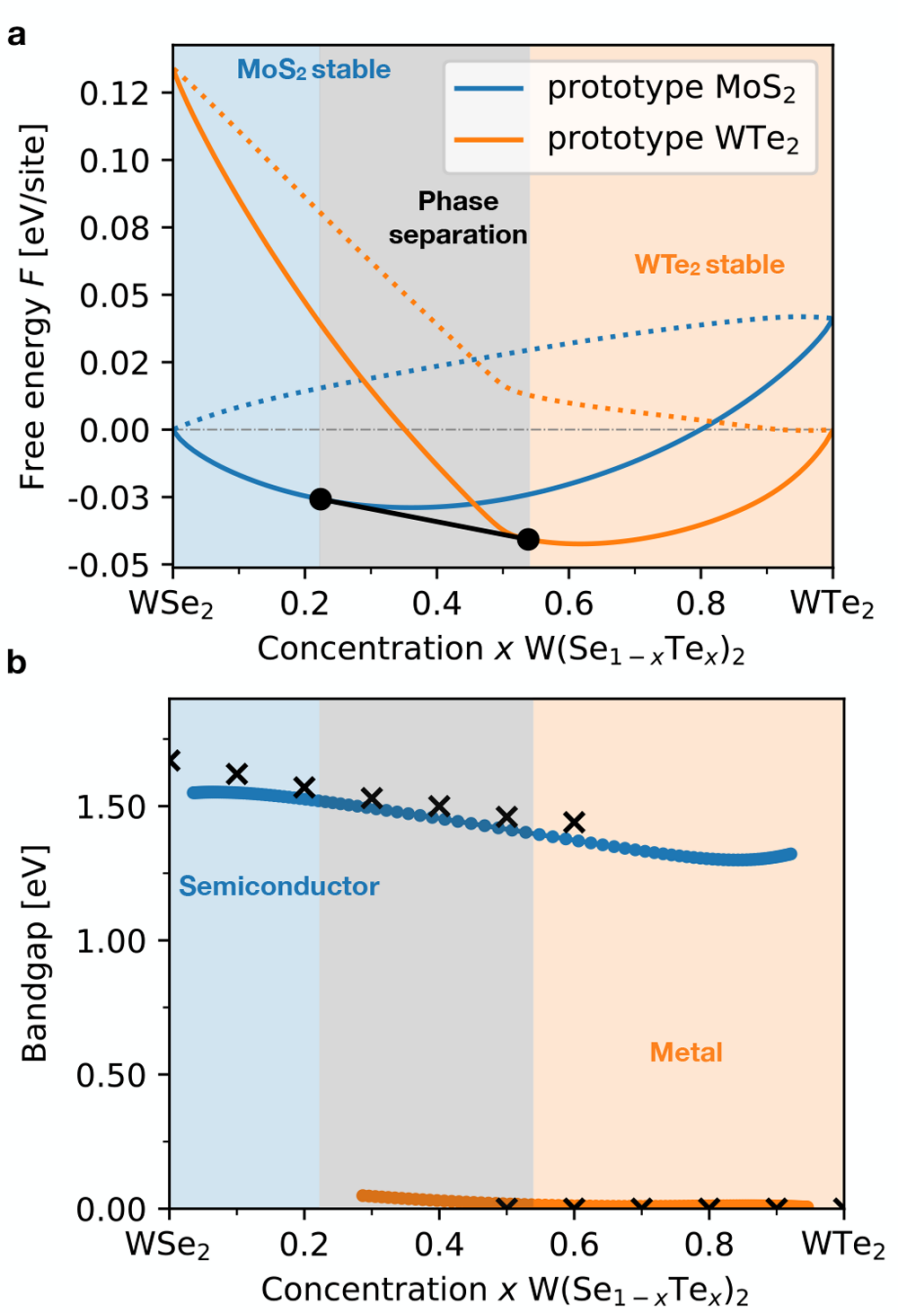
(a) Thermodynamic stability at finite temperature. The vertical axis reports the free energy *F* (in eV/lattice sites) at synthesis temperature *T* = 900 K^47^ against the concentration *x*. The free energy (solid lines) is estimated from the zero temperature convex hull (dotted lines) as outlined in the Methods. The solid black line between the dots shows the Maxwell construction defining the phase separating region (gray-shaded area) between the MoS_2_ (blue-shaded area) and WTe_2_ stable (orange-shaded area) ones. (b) Bandgap as a function of concentration in the MoS_2_ (blue line) and WTe_2_ (orange line line) prototypes. The shaded area marks the pMoS_2_-stable, phase-separating, and pWTe_2_-stable regions defined in (a). Black crosses report the experimental values adapted from ref (47).

Finally, we discuss in detail an example of alloying on the chalcogen site, which is predicted to show polymorphism. We focus on W(Se:Te)_2_, where polymorphism should occur between the GS of WSe_2_ (p-MoS_2_ prototype) and the GS of WTe_2_ (p-WTe_2_ prototype), as this will allow us to compare directly with experimental data on phase stability and optoelectronic properties. Figure 6d reports the formation energy of ordered configurations, which in this case is given by
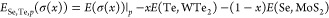
5At zero temperature the system is weakly phase separating in the p-MoS_2_ prototype (blue triangles in Figure 6d). There is a small interhost miscibility window on the Te-rich side in the p-WTe_2_ prototype (orange crosses Figure 6d): around *x* ≈ 0.9 an alloy in the p-WTe_2_ prototype is more stable than the phase-separation (see the black line falling below the dash-dotted gray line in Figure 6d). In order to compare with experiments, we estimate the finite temperature phase diagram with an approximated Boltzmann sampling; see Methods for details. Figure 7a reports the free energy of each prototype as a function of concentration at *T* = 900 K, compatible with CVD synthesis.^47^ Using the Maxwell construction, we estimate miscibility in the p-MoS_2_ prototype up to *x* = 0.22 (blue-shaded area in Figure 7a), phase separation in the range *x* ∈ [0.22, 0.54] (gray-shaded area), and miscibility in the p-WTe_2_ prototype from *x* = 0.54 (orange-shaded area). The estimated phase behavior is in agreement with the experiments in ref (47), which reports a phase transition in this pseudobinary system around *x* ≈ 0.5.

The comparison with experiments can be extended to optoelectronic properties. Figure 7b reports the bandgap in p-MoS_2_ (blue dots) and p-WTe_2_ (orange dots) prototypes as a function of concentration. See SI Section VII.B for details. The plot is divided in the three phase regions defined in Figure 7a. W(Se:Te)_2_ in the p-MoS_2_ prototype is a semiconductor with a bandgap decreasing from 1.55 to 1.30 eV as a function of concentration, while W(Se:Te)_2_ in the p-WTe_2_ prototype is a semimetal with a vanishingly small bandgap of 0.05 eV at *x* ≈ 0.5 that closes for *x* > 0.6. These results are in remarkable agreement with experiments (black crosses in Figure 7b): the CVD-grown samples in ref (47) are semiconductors with bandgaps around 1.5 eV up to *x* ≈ 0.6 and turn metallic for higher-concentrations, once the p-WTe_2_ geometry is more stable.

## VI Conclusions

We presented a systematic analysis of possible substitutional alloys in two-dimensional TMDs on both metal and chalcogenide sites. The best structural prototypes for alloying are identified via a ranking of a metastability metric. This ranking, visualized by the chemical space maps shown in Figures 4 and 5, provides a guideline for experimental synthesis and an assessment of thermodynamic stability for computational screening of properties of different compounds.

Predictions of phase separating and miscible systems by the metastability metric are in good agreement with experimental reports in the literature and with the systematic computational samplings of ordered structures carried out in this study for selected binary alloys from First-Principles. While this work focused on TMDs, the methodology developed here can be transferred to any stochiometry and composition, with the caveat that different systems might require a different underlying DFT protocol, e.g., Hubbard corrections for oxides.

The Pettifor maps of optimal prototype in Figures 4 and 5 can help to identify viable alloy candidates, minimizing the trial-and-error attempts and speeding up the discovery of novel materials for nanotechnology. In particular, these maps could aid CVD synthesis of novel ML alloys in non-native geometries that exhibit desirable properties.

In a wider context, the framework developed here fits in the effort of making chemical intuition quantitative. The exploration of a large data set, easily produced with modern DFT methods, allows us to rationalize trends across the periodic table and refine known empirical rules or adapt them to new chemical spaces. Here we showed how the evolution from more ionic bonds in sulfides to more covalent ones in tellurides results in more possibilities for alloying on the metal site. The analysis at fixed metal and varying chalcogenide confirms the chemical intuition that coordination is dictated by the *d* manifold of the metal, resulting in the dominance of the same GS prototype for sulfides, selenides, and tellurides. But, our quantitative analysis identifies cases that break this rule and where interesting polymorphism may be found. These trends are made quantitative by generalized Hume–Rothery rules and the metastability metric, resulting in the compact tool of the Pettifor maps for substitutional alloys in Figures 4 and 5.

To summarize, we presented a set of tools and ideas that will hopefully prove a useful guide for computational chemists and experimentalists while maping out the under-explored chemical space of two-dimensional TMDs.

## VII Methods

### VII.A Ab Initio Calculations

The total energy calculations are carried out with the Vienna *Ab Initio* Simulation Package (VASP),^57−59^ within the PAW framework for pseudopotentials.^60^ The generalized-gradient-approximation to DFT as parametrized by Perdew, Burke, and Ernzerhof^61^ was used in this work. The Kohn–Sham orbitals are expanded in a plane-wave basis with a cutoff of *E*_cutoff_ = 650 eV, and the BZ is sampled with a 17 × 17 × 1 mesh. The electronic density was computed self-consistently until the variation was below the threshold of 1 × 10^–6^ eV. We perform a spin-polarized calculation; the electronic structure can converge to nonmagnetic or ferromagnetic states, as we consider only primitive unit cells in our calculations. For lattice stability calculations, the positions of the ions in the unit cell were relaxed until the residual forces were below the threshold 1 × 10^–2^ eV/Å. For configurational sampling calculations, the positions of the ions and the unit cell were relaxed until the residual forces were below the threshold 1 × 10^–2^ eV/Å. To ensure no spurious interactions between the periodic images, a vacuum of 20 Å was added along the *c* axis.

Note that while error cancellation in the stoichiometric analysis carried out here makes the Hubbard U correction not necessary, ref (35) shows that this becomes fundamental in modeling thermochemical reactions involving valence changes, as the reaction enthalpy of most sulfurization reactions is not correctly described at U = 0.

### VII.B Approximated Boltzmann Sampling

A Boltzmann weighting of computed configurations
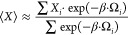
with Ω_*i*_ = *E*_*i*_ – *x*_*i*_·μ was used to estimate ensemble averages. In this approach, β is a parameter larger than 1/*kT* to compensate for the overweighting of high energy configurations implied by sampling over only a small part of the configurational space. The parameter β was chosen such that ⟨*E*⟩ closely resembles the convex hulls to reflect the expected small dependence of the internal energy on temperature common for solids. See the SI Section VII for more detail.

## Notes

The data underlying this study are available in the published article and its online supplementary material.

## Supporting Information Available

Details on database filtering, chemical and coordination space, ranking function, and configurations sampling, complete tables of lattice stability, metastability matrices of prototypes, mismatch matrices, and optimal prototype matrices (PDF)
Pristine compounds data (energies and structures) as a JSON database, lattice stability, lattice mismatch, metastability metric, and optimal prototype matrices provided in NumPy machine format, configurational sampling data (energies, structures, and similarity measure) included as an archive, and Python scripts to compute the metastability metric (and related quantities) and structural similarity (ZIP)
