# Supplementary material for: Design Guidelines for Two-Dimensional Transition Metal Dichalcogenide Alloys
Source: Chem Mater. 2022 Nov 29;34(23):10279–90. doi: 10.1021/acs.chemmater.2c01390 (PMC9753562; doi:10.1021/acs.chemmater.2c01390)
Supplement: Supplementary file 1 — cm2c01390_si_001.pdf [file cm2c01390_si_001.pdf]

Supplementary Material for Design Guidelines for Two-dimensional Transition Metal Dichalcogenide Alloys

Andrea Silva,[1,2,*] Jiangming Cao,[3] Tomas Polcar,[1,4] and Denis Kramer[1,3,5,†]

[1]*Engineering and Physical Sciences, University of Southampton, UK*

[2]*National Centre for Advanced Tribology Study, University of Southampton, UK*

[3]*Faculty of Mechanical and Civil Engineering, Helmut-Schmidt-University, Hamburg, Germany*

[4]*Advanced Materials Group, Department of Control Engineering, Faculty of Electrical Engineering, Czech Technical University in Prague (CTU), Czech Republic*

[5]*Department of Heterogeneous Catalysis, Helmholtz-Zentrum Hereon, Max-Planck-Strasse 1, Geesthacht, Germany*

CONTENTS

I. Details on database filtering, chemical and coordination space 3

II. Lattice stability tables 7

III. Metastability matrices of prototypes 12

IV. Mismatch 17

V. Ranking Function Details 22

1. Weight Selection for Optimal Host Matrix 22

VI. Optimal Prototype Matrix Details 24

VII. Configurations sampling details 29

A. $(Mo:Ti)Te_2$ details 30

B. $W(Se:Te)_2$ details 33

References 34

* a.silva@soton.ac.uk

† d.kramer@hsu-hh.de

I. DETAILS ON DATABASE FILTERING, CHEMICAL AND COORDINATION SPACE

The 2D material database from Mounet and coworkers [1] is based on structures in the ICSD [2] and COD [3] databases and contains unaries, binaries, ternaries, etc, spanning the whole periodic table. For this study, binaries MX_2 containing the elements in Table SI are selected from the database. The element used in the filters go beyond the TMD chemistry as layered geometry potentially relevant for TMDs may be expressed in the database with elements outside the TMD family, e.g. $NbTe_2$ prototypes in Table SII. The selection yields the 61 materials reported in Table SII.

Anions	H, N, O, F, P, S, Cl, As, Se, Br, Te, I
Cations	Ti, V, Cr, Mn, Fe, Co, Ni, Zr, Nb, Mo, Tc, Ru, Rh, Pd, Hf, Ta, W, Re, Os, Ir, Pt

Table SI: Anion (top row) and cation (bottom row) elements.

Prototype	Formula	Lattice spacing [Å]	DB key (DB name)
CdI_2			
	$CoBr_2$	3.729	9016149 (COD)
	$CoCl_2$	3.528	9014719 (COD), 9009126 (COD)
	CoI_2	6.715	9009100 (COD)
	CoO_2	2.813	1522027 (COD)
	$CrSe_2$	5.668	626718 (ICSD), 9012110 (COD)
	$FeBr_2$	3.730	9009102 (COD)
	$FeCl_2$	3.405	9014952 (COD), 9009128 (COD), 9015767 (COD)
	FeI_2	4.019	9009103 (COD)
	HfS_2	3.630	638847 (ICSD)
	$HfSe_2$	3.764	182678 (ICSD)
	$HfTe_2$	3.970	603713 (ICSD)
	$MnBr_2$	6.712	67500 (ICSD), 9009109 (COD)
	$MnCl_2$	6.379	9009130 (COD)
	MnI_2	4.129	9009110 (COD)

MoS_2	3.187	26622 (ICSD)
NbS_2	3.373	24755 (ICSD), 7204814 (COD)
$NbSe_2$	3.476	76576 (ICSD), 76577 (ICSD), 645377 (ICSD), 645379 (ICSD)
$NbTe_2$	3.670	645529 (ICSD)
$NiBr_2$	3.680	9008013 (COD)
$NiCl_2$	3.488	9009132 (COD)
NiI_2	3.956	9009133 (COD)
NiO_2	2.824	1522025 (COD), 9016308 (COD)
PtO_2	3.150	24923 (ICSD), 76431 (ICSD)
PtS_2	3.556	5910085 (COD)
$PtSe_2$	3.712	9009117 (COD)
TaS_2	3.377	9011539 (COD), 24757 (ICSD), 52115 (ICSD), 52117 (ICSD)
$TaSe_2$	3.498	651954 (ICSD), 24313 (ICSD), 24318 (ICSD), 1521538 (COD)
$TiBr_2$	3.504	26078 (ICSD)
$TiCl_2$	3.285	9009121 (COD)
TiS_2	3.412	1010275 (COD), 60015 (ICSD)
$TiSe_2$	3.536	1010276 (COD), 31343 (ICSD)
$TiTe_2$	3.764	1008063 (COD)
VBr_2	6.376	246906 (ICSD)
VCl_2	5.977	1528165 (COD)
VI_2	6.923	246907 (ICSD)
VS_2	3.184	651361 (ICSD), 68713 (ICSD)
VSe_2	3.339	86520 (ICSD)
VTe_2	3.635	603582 (ICSD)
ZrS_2	3.671	5910006 (COD)
$ZrSe_2$	3.798	5910026 (COD)
$ZrTe_2$	3.974	653213 (ICSD)

CrI_2			
	CrI_2	7.797	4073 (ICSD)
FeO_2			
	FeO_2	2.789	9015156 (COD)
MoS_2			
	MoS_2	3.188	644259 (ICSD), 9009148 (COD), 9007660 (COD)
	$MoSe_2$	3.316	644346 (ICSD), 1528933 (COD), 49800 (ICSD)
	$MoTe_2$	3.565	9009147 (COD)
	NbS_2	3.343	237034 (ICSD), 43697 (ICSD), 74702 (ICSD)
	$NbSe_2$	3.466	9014575 (COD), 2310533 (COD), 2310534 (COD), 645383 (ICSD), 76577 (ICSD), 645377 (ICSD), 645379 (ICSD), 53102 (ICSD), 640056 (ICSD), 26288 (ICSD), 26287 (ICSD)
	$ReSe_2$	3.456	650091 (ICSD)
	TaS_2	3.337	651083 (ICSD), 9007815 (COD), 52117 (ICSD), 640379 (ICSD)
	$TaSe_2$	3.467	651956 (ICSD), 24315 (ICSD), 24316 (ICSD), 26249 (ICSD), 2310532 (COD), 24318 (ICSD), 1521538 (COD)
	WS_2	3.187	9012192 (COD), 9009145 (COD)
	WSe_2	3.317	9012193 (COD)
	WTe_2	3.567	653170 (ICSD)
	$ZrCl_2$	3.407	30052 (ICSD)
$NbTe_2$			
	$CrBr_2$	6.549	23903 (ICSD)
$PdCl_2$			
	$PdCl_2$	4.249	421221 (ICSD)
PdS_2			
	PdS_2	5.463	2310589 (COD)
WTe_2			
	$MoTe_2$	3.462	2310356 (COD)
	WTe_2	3.500	2310355 (COD)

ZrI_2	3.766	26418 (ICSD), 24807 (ICSD)

Table SII: Binaries from the 2D database [1] containing the elements in Table SI. The first column reports the prototype reported in [1], the second column reports the chemical formula of the compound, the third column shows the lattice parameter reported in [1] and the fourth column the entries in the ICSD and COD databases associated with the compound.

These selected compounds appear in the eight prototypes reported in Table SIII, where the second column reports the space group defining the symmetry of each prototype geometry, shown in Figure 1a-h in the main text.

Prototype	Space group (number)
CdI_2	$P\bar{3}m1$ (164)
CrI_2	$P2_1/m$ (11)
FeO_2	Pmmn (59)
MoS_2	$P\bar{6}m2$ (187)
$NbTe_2$	$P\bar{1}$ (2)
$PdCl_2$	$P2_1/c$ (14)
PdS_2	$P2_1/c$ (14)
WTe_2	$P2_1/m$ (11)

Table SIII: Space group of the considered prototypes, extracted from Ref. [1]. The symmetry was computed using the Phonopy suite[4] using a symmetry tolerance of 1×10^{-2} Å.

The chemical and geometry space of this study is obtained by placing, with a combinatorial process, a TMs in Table SI on the cation sites, a chalcogenides (S, Se, Te) on anion sites of one of the identified prototype geometries in Table SIII. This combinatorial process yields the 504 materials whose lattice stability is reported in the matrices in Figure 2 in the main text and below. The equilibrium configuration of each material is obtained from EoS

calculations.

II. LATTICE STABILITY TABLES

Numerical values of the matrices shown in Figure 2 in the main text.

S-Proto	CrI_2	CdI_2	$PdCl_2$	MoS_2	PdS_2	FeO_2	WTe_2	$NbTe_2$
Ti	0.168	0	0.204	0.409	1.27	0.325	0.0169	0.29
V	0.223	0.0466	0.286	0	1.33	0.421	0.0854	0.344
Cr	0.364	0.402	0.459	0	1.39	0.666	0.336	0.507
Mn	0.118	0	0.176	0.31	1.02	0.532	0.0127	0.206
Fe	0.0758	0.0314	0.143	0.202	0.675	0.425	0	0.159
Co	0.158	0.0926	0.138	0.443	0.489	0.49	0.0737	0
Ni	0.256	0.0989	0.302	0.553	0	0.652	0.116	0.36
Zr	0.208	0	0.227	0.561	1.38	0.273	0.0208	0.375
Nb	0.289	0.0955	0.343	0	1.39	0.709	0.117	0.444
Mo	0.644	0.837	1	0	1.77	1.67	0.549	1.26
Tc	0.0735	0.666	0.287	0.351	1.14	0.977	0	0.391
Ru	0.34	0.669	0	0.725	1.19	0.705	0.0695	0.211
Rh	0.303	0.276	0.133	0.944	0.561	0.594	0.227	0
Pd	0.457	0.313	0.5	1.02	0	0.975	0.33	0.567
Hf	0.223	0	0.243	0.642	1.61	0.329	0.0223	0.405
Ta	0.267	0.0614	0.318	0	1.61	0.76	0.0861	0.432
W	0.679	0.893	0.955	0	1.88	1.81	0.537	1.32
Re	0.11	0.827	0.326	0.647	1.55	1.21	0	0.425
Os	0.497	0.81	0	1.05	1.6	0.889	0.22	0.28
Ir	0.391	0.278	0.209	1.4	0.977	0.662	0.249	0
Pt	0.206	0	0.26	1.81	0.158	0.808	0.0325	0.356

Se-Proto	CrI_2	CdI_2	$PdCl_2$	MoS_2	PdS_2	FeO_2	WTe_2	$NbTe_2$
Ti	0.135	0	0.176	0.343	0.999	0.401	0.0154	0.217
V	0.154	0.0478	0.207	0	0.87	0.447	0.0632	0.263
Cr	0.184	0.104	0.173	0	1	0.414	0.163	0.222

Mn	0.096	0	0.174	0.397	1.04	0.502	0.0102	0.167
Fe	0.0678	0.0363	0.145	0.167	0.887	0.466	0	0.145
Co	0.123	0.0453	0.122	0.366	0.756	0.495	0.0443	0
Ni	0.118	0	0.186	0.473	0.159	0.428	0.0168	0.191
Zr	0.185	0	0.208	0.43	1.02	0.359	0.0185	0.329
Nb	0.27	0.0974	0.316	0	0.662	0.844	0.115	0.406
Mo	0.39	0.705	0.742	0	1.04	1.53	0.33	0.819
Tc	0.0345	0.619	0.186	0.323	0.777	1.07	0	0.298
Ru	0.326	0.642	0	0.632	1.09	0.818	0.0686	0.211
Rh	0.275	0.245	0.14	0.728	0.921	0.599	0.208	0
Pd	0.195	0.0668	0.209	0.78	0	0.619	0.0447	0.288
Hf	0.202	0	0.227	0.518	1.24	0.402	0.02	0.362
Ta	0.253	0.0691	0.3	0	0.818	0.88	0.0892	0.396
W	0.379	0.773	0.687	0	0.244	1.6	0.267	0.793
Re	0.0815	0.786	0.191	0.579	1.06	1.31	0	0.299
Os	0.511	0.836	0	0.906	1.53	1	0.202	0.267
Ir	0.386	0.34	0.181	1.11	1.29	0.725	0.304	0
Pt	0.175	0	0.199	1.36	0.321	0.698	0.0183	0.31

Te-Proto	CrI_2	CdI_2	$PdCl_2$	MoS_2	PdS_2	FeO_2	WTe_2	$NbTe_2$
Ti	0.0745	0	0.128	0.302	0.756	0.471	0.0108	0.133
V	0.017	0.0293	0.105	0.0239	0.623	0.436	0	0.148
Cr	0.045	0	0.0973	0.247	0.847	0.384	0.239	0.0752
Mn	0.0888	0	0.15	0.268	0.858	0.406	0.0194	0.131
Fe	0.0655	0.0577	0.16	0.116	0.881	0.408	0	0.139
Co	0.0844	0	0.141	0.251	0.888	0.419	0.0111	0.0576
Ni	0.086	0	0.161	0.25	0.306	0.275	0.00866	0.149
Zr	0.12	0	0.175	0.293	0.676	0.457	0.0136	0.19
Nb	0.059	0.025	0.223	0	0.422	0.811	0.0361	0.218
Mo	0.0714	0.515	0.413	0	0.671	1.24	0.0439	0.9
Tc	0.0446	0.656	0.0973	0.328	0.692	1.05	0	0.232

Ru	0.309	0.577	0	0.497	0.918	0.781	0.0958	0.192
Rh	0.165	0.107	0.154	0.41	0.961	0.525	0.113	0
Pd	0.104	0	0.154	0.411	0.0297	0.367	0.0103	0.16
Hf	0.142	0	0.174	0.382	0.9	0.469	0.0165	0.24
Ta	0.0103	0.0167	0.22	0	0.54	0.832	0.0435	0.258
W	0.0819	0.654	0.384	0.0881	0.923	1.41	0	0.481
Re	0.0631	0.843	0.0181	0.439	0.946	0.756	0	0.17
Os	0.513	0.682	0	0.662	1.41	0.886	0.17	0.232
Ir	0.272	0.204	0.193	0.69	1.4	0.645	0.209	0
Pt	0.179	0	0.227	0.899	0.374	0.533	0.0162	0.305

Table SIV: Lattice stability in eV/site at fixed chalcogenide (indicated in the header line) and varying metal (first column).

Ti-proto	CrI_2	CdI_2	$PdCl_2$	MoS_2	PdS_2	FeO_2	WTe_2	$NbTe_2$
S	0.168	0	0.204	0.409	1.27	0.325	0.0169	0.29
Se	0.135	0	0.176	0.343	0.999	0.401	0.0154	0.217
Te	0.0745	0	0.128	0.302	0.756	0.471	0.0108	0.133
V-proto	CrI_2	CdI_2	$PdCl_2$	MoS_2	PdS_2	FeO_2	WTe_2	$NbTe_2$
S	0.223	0.0466	0.286	0	1.33	0.421	0.0854	0.344
Se	0.154	0.0478	0.207	0	0.87	0.447	0.0632	0.263
Te	0.017	0.0293	0.105	0.0239	0.623	0.436	0	0.148
Cr-proto	CrI_2	CdI_2	$PdCl_2$	MoS_2	PdS_2	FeO_2	WTe_2	$NbTe_2$
S	0.364	0.402	0.459	0	1.39	0.666	0.336	0.507
Se	0.184	0.104	0.173	0	1	0.414	0.163	0.222
Te	0.045	0	0.0973	0.247	0.847	0.384	0.239	0.0752
Mn-proto	CrI_2	CdI_2	$PdCl_2$	MoS_2	PdS_2	FeO_2	WTe_2	$NbTe_2$
S	0.118	0	0.176	0.31	1.02	0.532	0.0127	0.206
Se	0.096	0	0.174	0.397	1.04	0.502	0.0102	0.167
Te	0.0888	0	0.15	0.268	0.858	0.406	0.0194	0.131

Fe-proto	CrI_2	CdI_2	$PdCl_2$	MoS_2	PdS_2	FeO_2	WTe_2	$NbTe_2$
S	0.0758	0.0314	0.143	0.202	0.675	0.425	0	0.159
Se	0.0678	0.0363	0.145	0.167	0.887	0.466	0	0.145
Te	0.0655	0.0577	0.16	0.116	0.881	0.408	0	0.139
Co-proto	CrI_2	CdI_2	$PdCl_2$	MoS_2	PdS_2	FeO_2	WTe_2	$NbTe_2$
S	0.158	0.0926	0.138	0.443	0.489	0.49	0.0737	0
Se	0.123	0.0453	0.122	0.366	0.756	0.495	0.0443	0
Te	0.0844	0	0.141	0.251	0.888	0.419	0.0111	0.0576
Ni-proto	CrI_2	CdI_2	$PdCl_2$	MoS_2	PdS_2	FeO_2	WTe_2	$NbTe_2$
S	0.256	0.0989	0.302	0.553	0	0.652	0.116	0.36
Se	0.118	0	0.186	0.473	0.159	0.428	0.0168	0.191
Te	0.086	0	0.161	0.25	0.306	0.275	0.00866	0.149
Zr-proto	CrI_2	CdI_2	$PdCl_2$	MoS_2	PdS_2	FeO_2	WTe_2	$NbTe_2$
S	0.208	0	0.227	0.561	1.38	0.273	0.0208	0.375
Se	0.185	0	0.208	0.43	1.02	0.359	0.0185	0.329
Te	0.12	0	0.175	0.293	0.676	0.457	0.0136	0.19
Nb-proto	CrI_2	CdI_2	$PdCl_2$	MoS_2	PdS_2	FeO_2	WTe_2	$NbTe_2$
S	0.289	0.0955	0.343	0	1.39	0.709	0.117	0.444
Se	0.27	0.0974	0.316	0	0.662	0.844	0.115	0.406
Te	0.059	0.025	0.223	0	0.422	0.811	0.0361	0.218
Mo-proto	CrI_2	CdI_2	$PdCl_2$	MoS_2	PdS_2	FeO_2	WTe_2	$NbTe_2$
S	0.644	0.837	1	0	1.77	1.67	0.549	1.26
Se	0.39	0.705	0.742	0	1.04	1.53	0.33	0.819
Te	0.0714	0.515	0.413	0	0.671	1.24	0.0439	0.9
Tc-proto	CrI_2	CdI_2	$PdCl_2$	MoS_2	PdS_2	FeO_2	WTe_2	$NbTe_2$
S	0.0735	0.666	0.287	0.351	1.14	0.977	0	0.391
Se	0.0345	0.619	0.186	0.323	0.777	1.07	0	0.298
Te	0.0446	0.656	0.0973	0.328	0.692	1.05	0	0.232
Ru-proto	CrI_2	CdI_2	$PdCl_2$	MoS_2	PdS_2	FeO_2	WTe_2	$NbTe_2$
S	0.34	0.669	0	0.725	1.19	0.705	0.0695	0.211

Se	0.326	0.642	0	0.632	1.09	0.818	0.0686	0.211
Te	0.309	0.577	0	0.497	0.918	0.781	0.0958	0.192
Rh-proto	CrI_2	CdI_2	$PdCl_2$	MoS_2	PdS_2	FeO_2	WTe_2	$NbTe_2$
S	0.303	0.276	0.133	0.944	0.561	0.594	0.227	0
Se	0.275	0.245	0.14	0.728	0.921	0.599	0.208	0
Te	0.165	0.107	0.154	0.41	0.961	0.525	0.113	0
Pd-proto	CrI_2	CdI_2	$PdCl_2$	MoS_2	PdS_2	FeO_2	WTe_2	$NbTe_2$
S	0.457	0.313	0.5	1.02	0	0.975	0.33	0.567
Se	0.195	0.0668	0.209	0.78	0	0.619	0.0447	0.288
Te	0.104	0	0.154	0.411	0.0297	0.367	0.0103	0.16
Hf-proto	CrI_2	CdI_2	$PdCl_2$	MoS_2	PdS_2	FeO_2	WTe_2	$NbTe_2$
S	0.223	0	0.243	0.642	1.61	0.329	0.0223	0.405
Se	0.202	0	0.227	0.518	1.24	0.402	0.02	0.362
Te	0.142	0	0.174	0.382	0.9	0.469	0.0165	0.24
Ta-proto	CrI_2	CdI_2	$PdCl_2$	MoS_2	PdS_2	FeO_2	WTe_2	$NbTe_2$
S	0.267	0.0614	0.318	0	1.61	0.76	0.0861	0.432
Se	0.253	0.0691	0.3	0	0.818	0.88	0.0892	0.396
Te	0.0103	0.0167	0.22	0	0.54	0.832	0.0435	0.258
W-proto	CrI_2	CdI_2	$PdCl_2$	MoS_2	PdS_2	FeO_2	WTe_2	$NbTe_2$
S	0.679	0.893	0.955	0	1.88	1.81	0.537	1.32
Se	0.379	0.773	0.687	0	0.244	1.6	0.267	0.793
Te	0.0819	0.654	0.384	0.0881	0.923	1.41	0	0.481
Re-proto	CrI_2	CdI_2	$PdCl_2$	MoS_2	PdS_2	FeO_2	WTe_2	$NbTe_2$
S	0.11	0.827	0.326	0.647	1.55	1.21	0	0.425
Se	0.0815	0.786	0.191	0.579	1.06	1.31	0	0.299
Te	0.0631	0.843	0.0181	0.439	0.946	0.756	0	0.17
Os-proto	CrI_2	CdI_2	$PdCl_2$	MoS_2	PdS_2	FeO_2	WTe_2	$NbTe_2$
S	0.497	0.81	0	1.05	1.6	0.889	0.22	0.28
Se	0.511	0.836	0	0.906	1.53	1	0.202	0.267
Te	0.513	0.682	0	0.662	1.41	0.886	0.17	0.232

Ir-proto	CrI_2	CdI_2	$PdCl_2$	MoS_2	PdS_2	FeO_2	WTe_2	$NbTe_2$
S	0.391	0.278	0.209	1.4	0.977	0.662	0.249	0
Se	0.386	0.34	0.181	1.11	1.29	0.725	0.304	0
Te	0.272	0.204	0.193	0.69	1.4	0.645	0.209	0
Pt-proto	CrI_2	CdI_2	$PdCl_2$	MoS_2	PdS_2	FeO_2	WTe_2	$NbTe_2$
S	0.206	0	0.26	1.81	0.158	0.808	0.0325	0.356
Se	0.175	0	0.199	1.36	0.321	0.698	0.0183	0.31
Te	0.179	0	0.227	0.899	0.374	0.533	0.0162	0.305

Table SV: Lattice stability in eV/site at fixed metal (indicated in the header line) and varying chalcogenide (first column).

III. METASTABILITY MATRICES OF PROTOTYPES

Figures S1 to S3 report the metastability metric matrices for fix chalcogenide and Fig. S4 for fix metal. The prototype is reported in the title above the figure, while x and y axes show the varying element. Optimal prototype for TM pairs. The colourcode shows the energy cost at each end of the window, in eV/site. The scale is reported in the first colorbar on the right Fig. S16. The energy cost of the mark refers to the end of the metastability window closer to the MS_2 indexed by the x axis. The size of the marker encodes the metastability window size, as reported by the legend on the far right in Fig. S16. The edge color of each marker indicates whether the optimal prototype is the ground state of both (green), one (gray) or neither (red) the pristine TMDs comprising the $(M : Q)X_2$ mixture. Marker-prototype correspondence is reported in the legend at the bottom right. Markers on the diagonal show the GS prototype of the corresponding TMD.

As an example, consider metastability matrix within the MoS_2 host, Fig. S1. Regions of favourable metastability windows (large blue marks) can be seen around group V (V, Nb, Ta) and group VI (Cr, Mo, W) elements, which according to Fig. S1b prefer this coordination. This observation is in agreement with the experimental realisation of a few alloys of this type [5, 6]. A large energy penalty (red marks) is associated with group VI and group X (Ni, Pd, Pt) transition metal mixtures, e.g. $Mo_xPd_{1-x}S_2|_{MoS_2}$, indicating MoS_2 prototype

as a poor candidate for mixing. Indeed, only Ni-doped MoS_2 MLs with isolated impurities are reported by experiments [7], and Ni shows the lowest mixing energy with MoS_2 among group X elements. Finally, a narrow metastability window (small marks) suggest that other prototypes are preferred for the corresponding mixture, even when MoS_2 is the prototype of one TM; an example of this is $Ti_xTa_{1-x}S_2|_{MoS_2}$, which favours the CdI_2 prototype, as shown below.

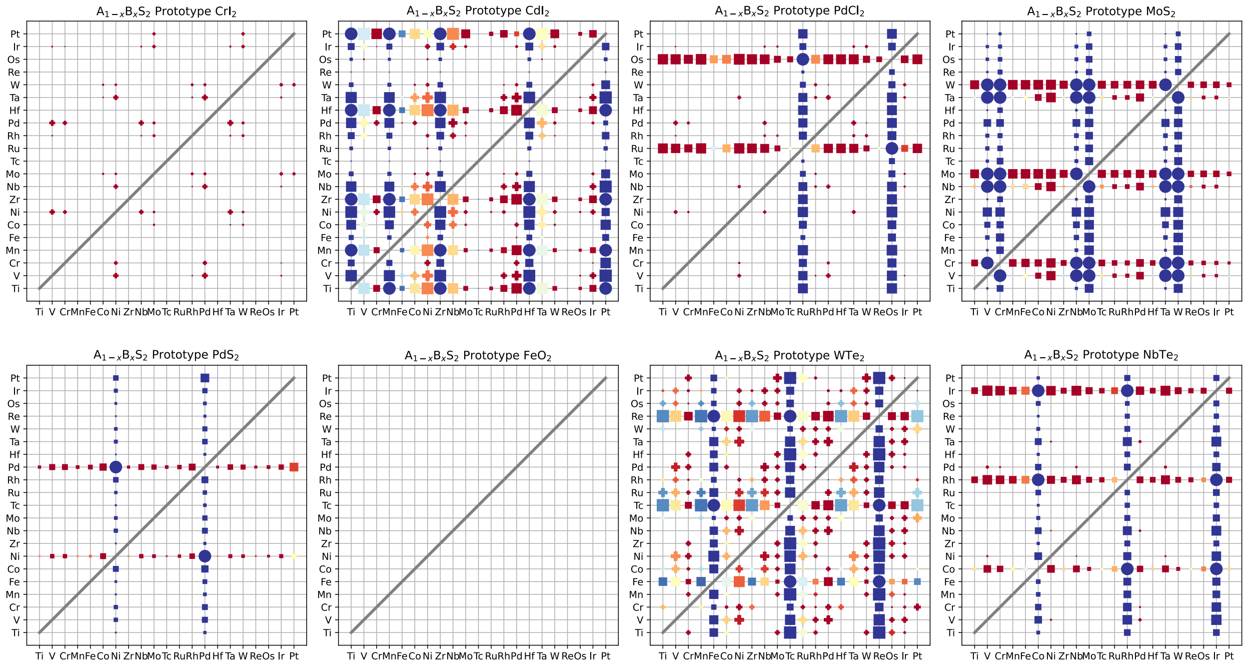

Figure S1: Metastability metric at fix chalcogenide: S

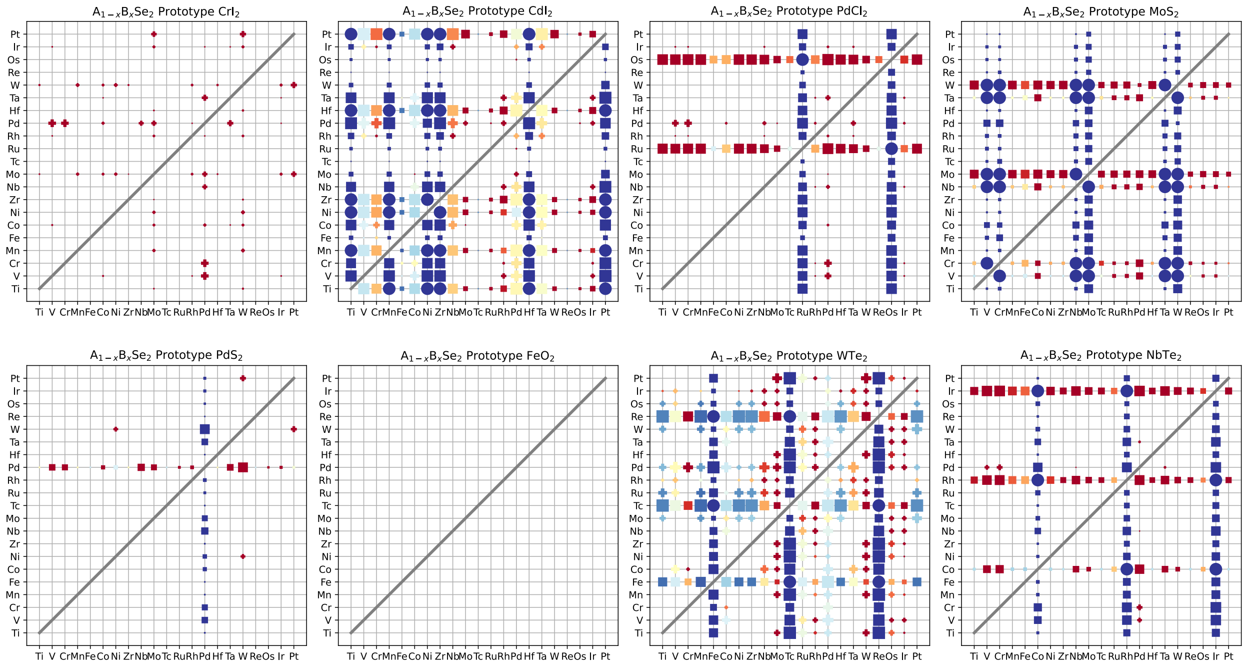

Figure S2: Metastability metric at fix chalcogenide: Se

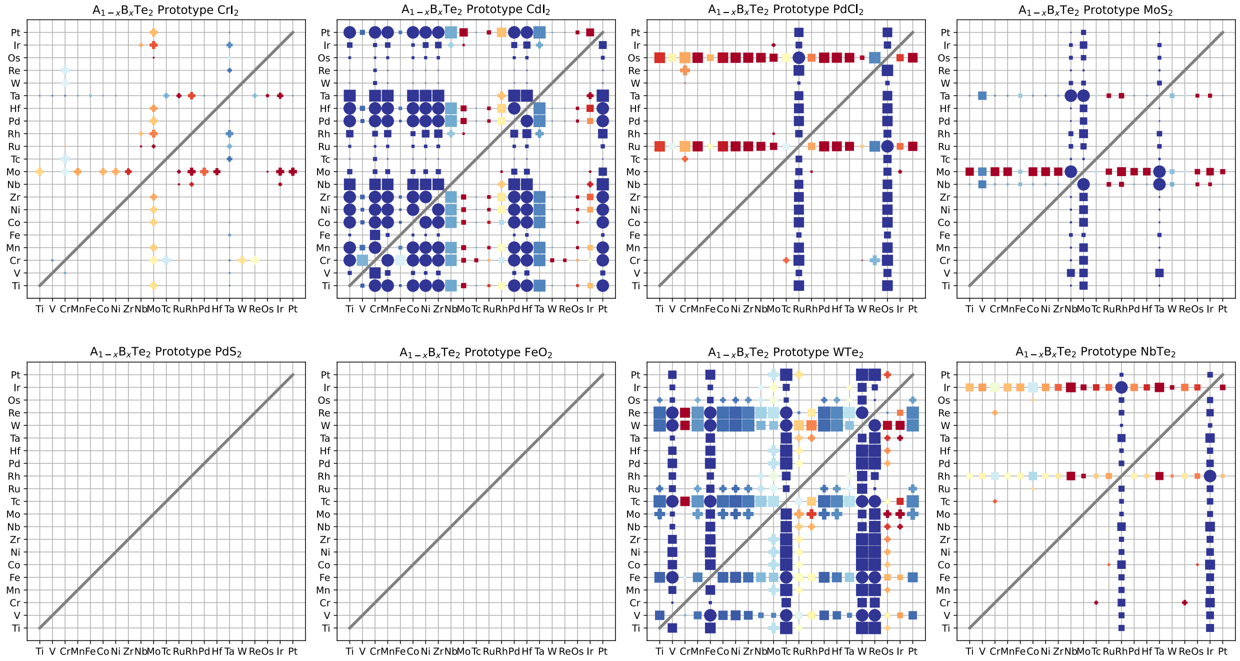

Figure S3: Metastability metric at fix chalcogenide: Te

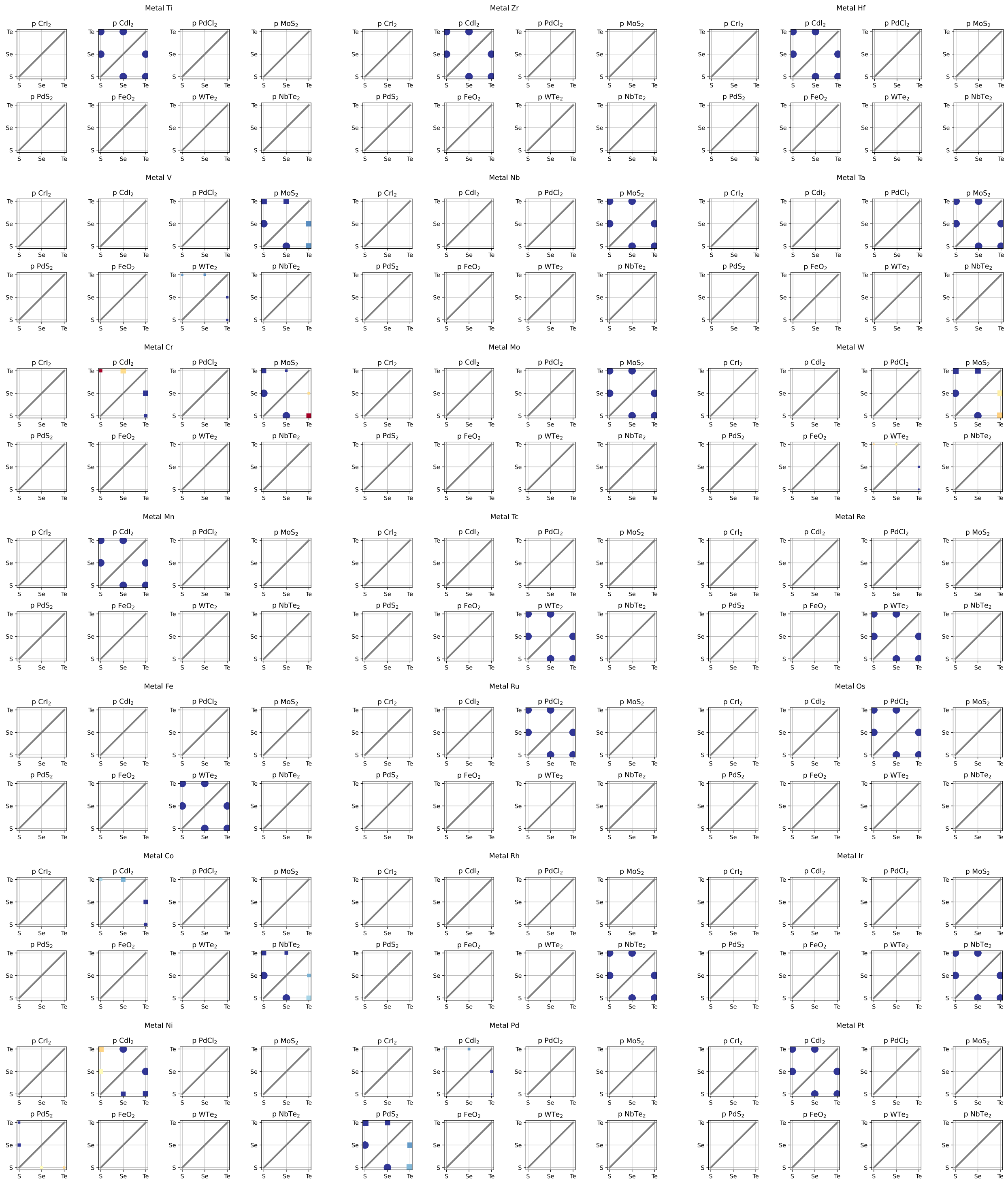

Figure S4: Metastability metric at fix TM

IV. MISMATCH

Figures S5 and S6 reports the lattice parameters l of the MX_2 binaries. For hexagonal cells, the table reports the magnitude of the in-plane lattice vector. For orthorombic ones, the table reports the smallest in-plane lattice vector of the unit cell.

Figures S7 to S10 report the mismatch $\delta_{ij} = |1 - l_i/l_j|$ for all transition metals combinations within the prototype p specified above the plot. In each plot, a blue edge highlights the elements with $\delta \leq 0.15$, while a red edge highlights $\delta > 0.15$.

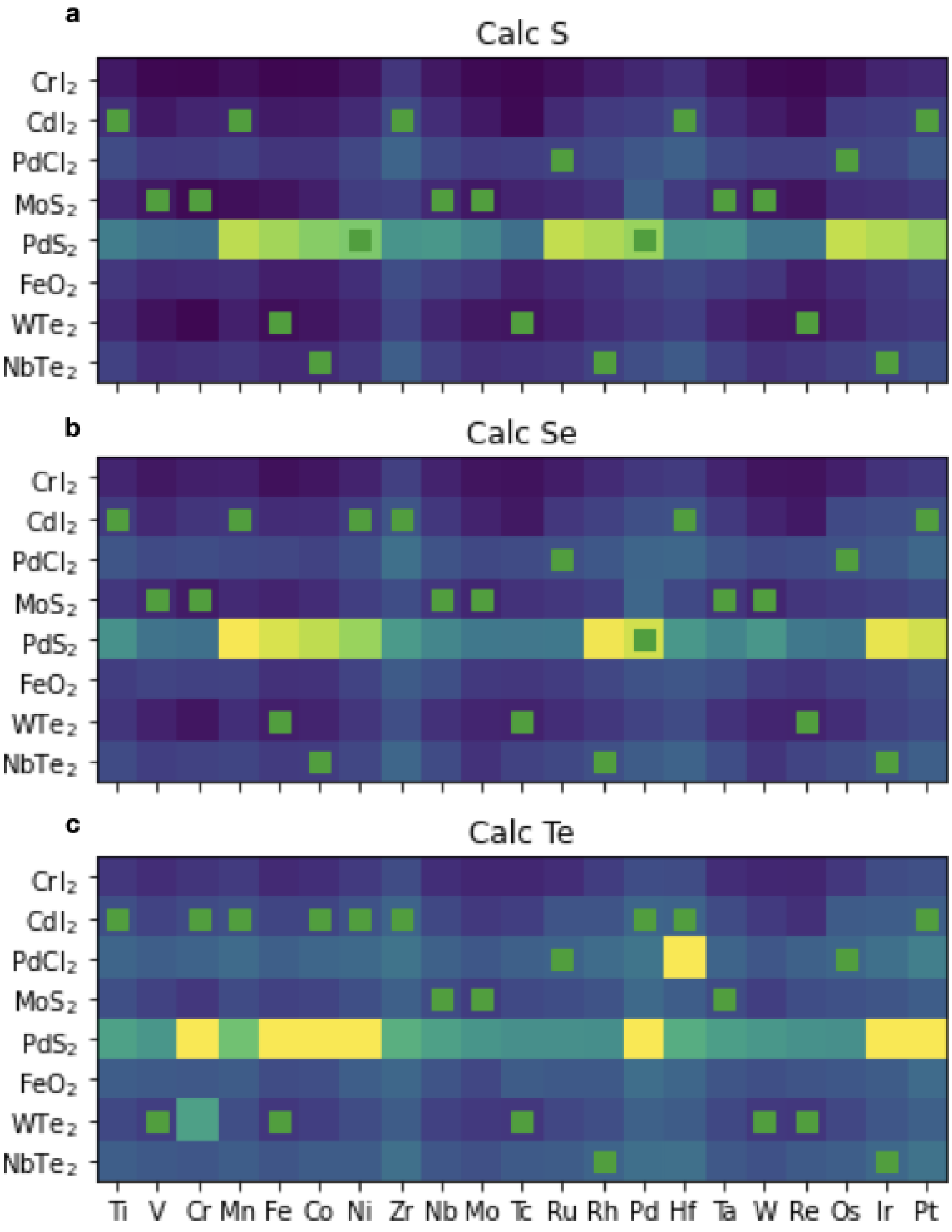

Figure S5: Lattice parameters for fixed chalcogenide.

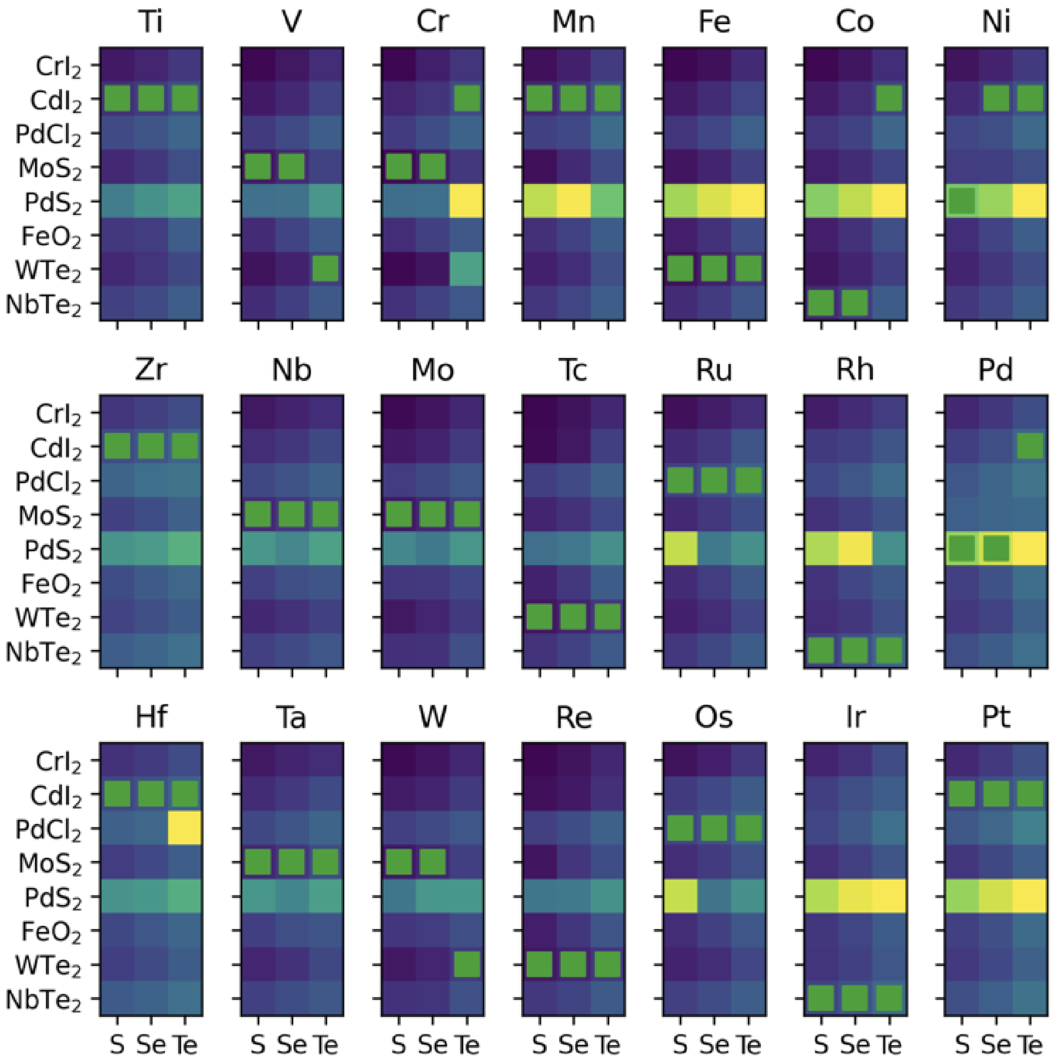

Figure S6: Lattice parameters for fixed metal.

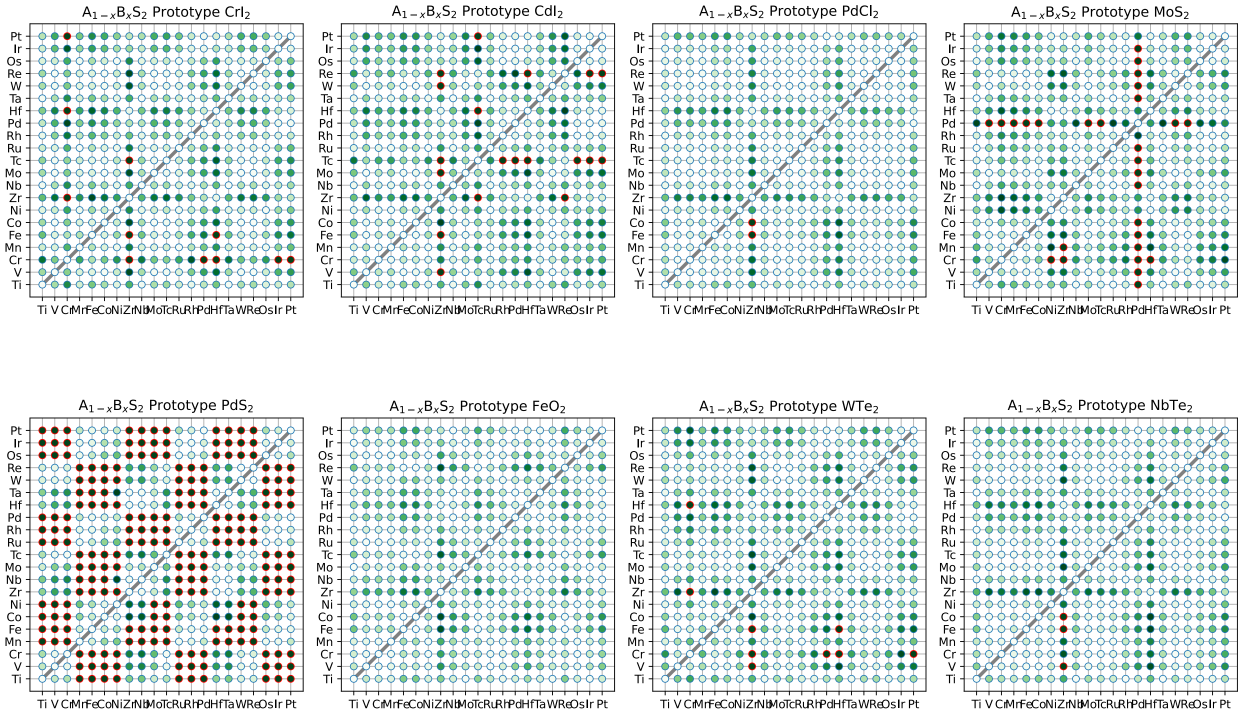

Figure S7: Mismatch at fix chalcogenide: S

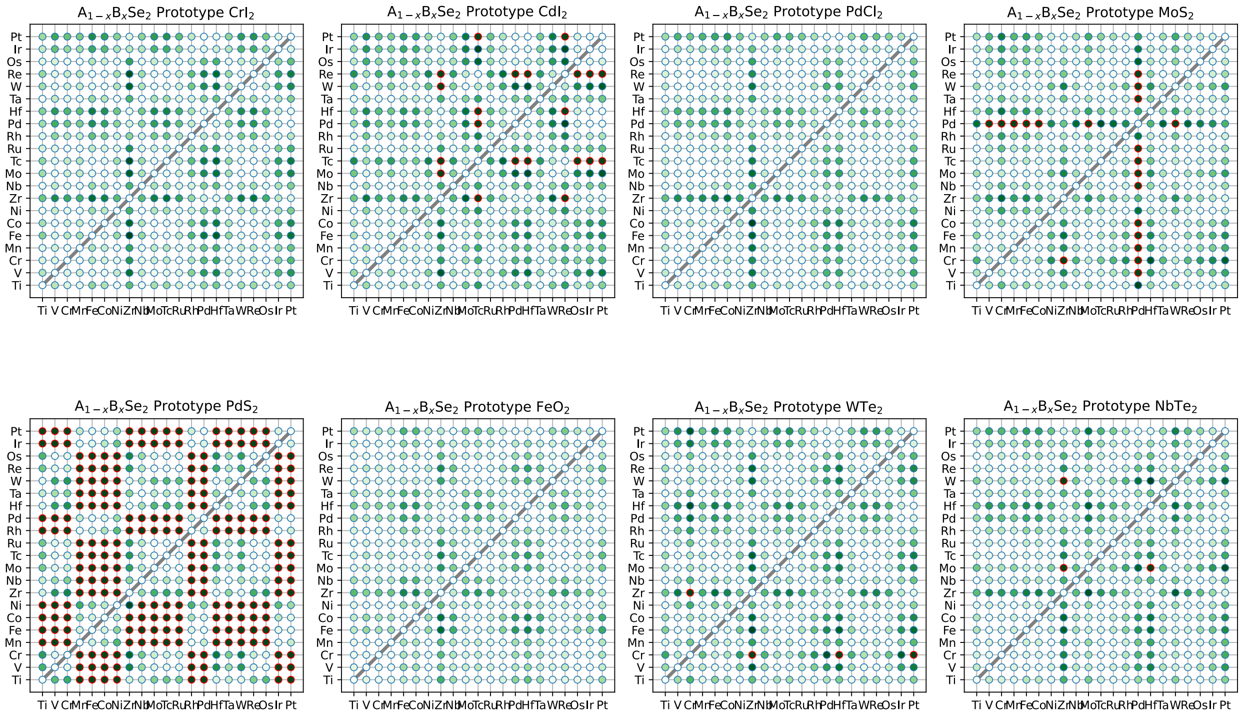

Figure S8: Mismatch at fix chalcogenide: Se

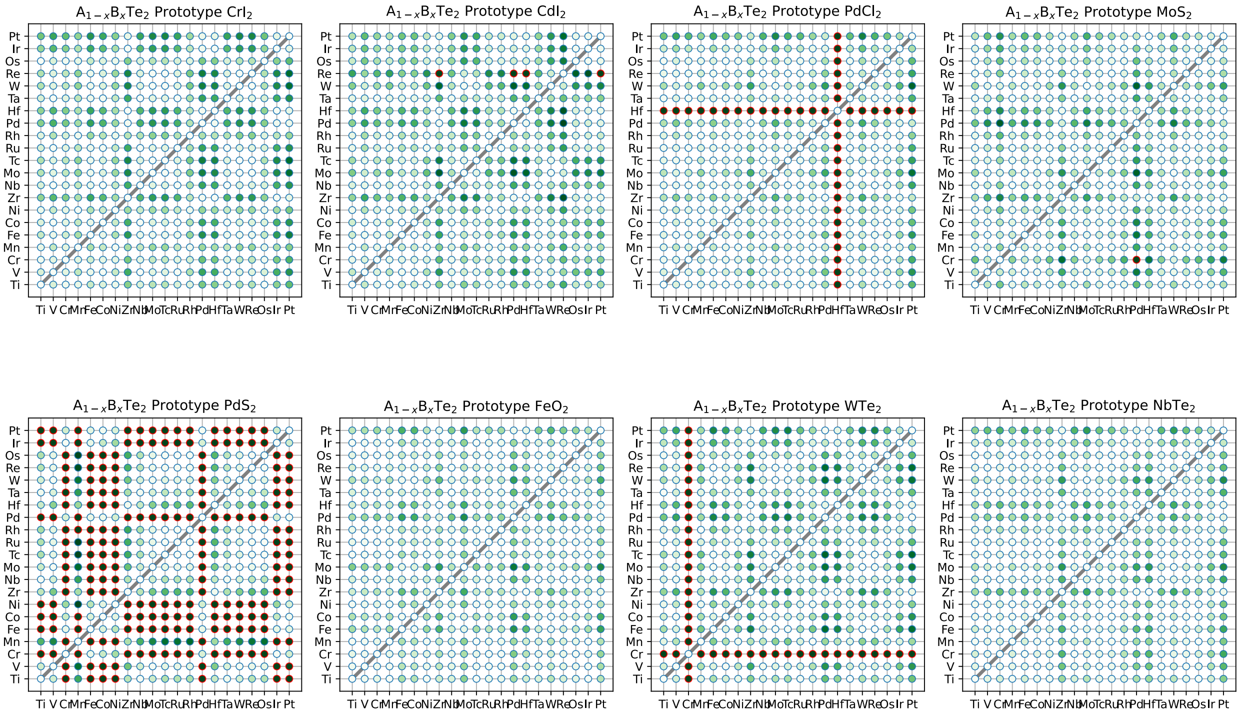

Figure S9: Mismatch at fix chalcogenide: Te

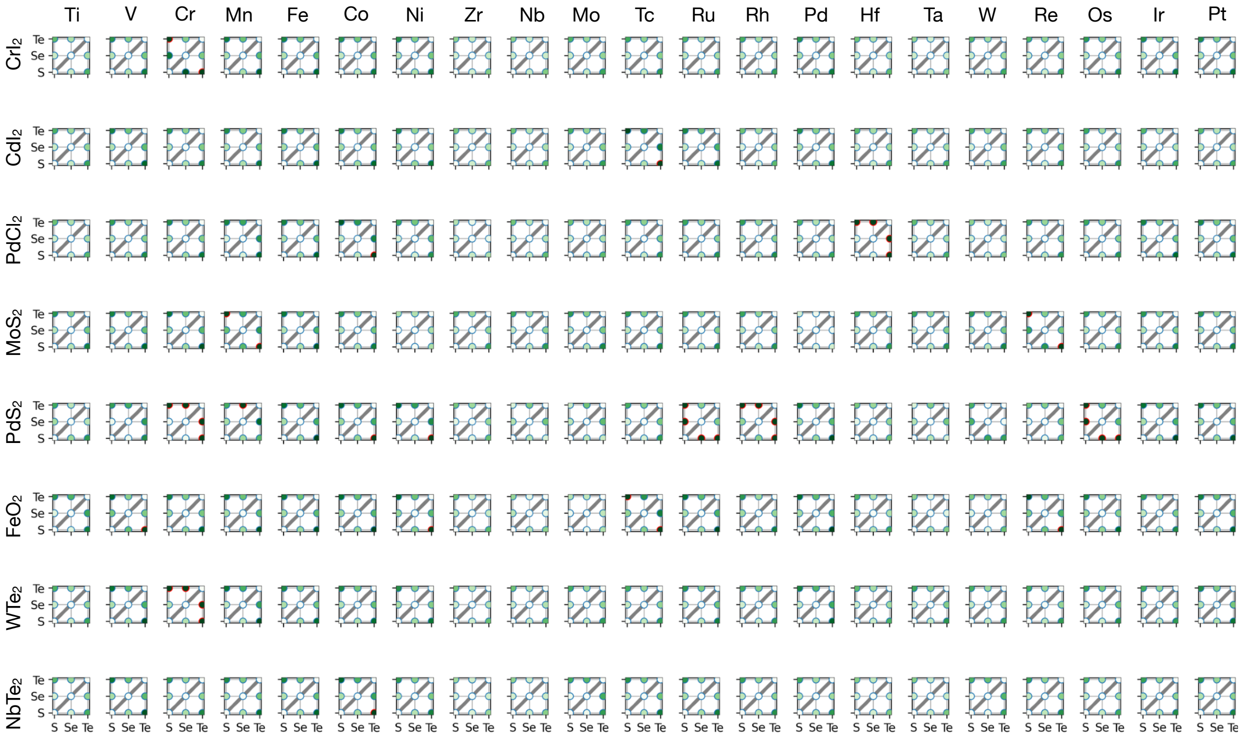

Figure S10: Mismatch at fix metals, all chalcogenides

V. RANKING FUNCTION DETAILS

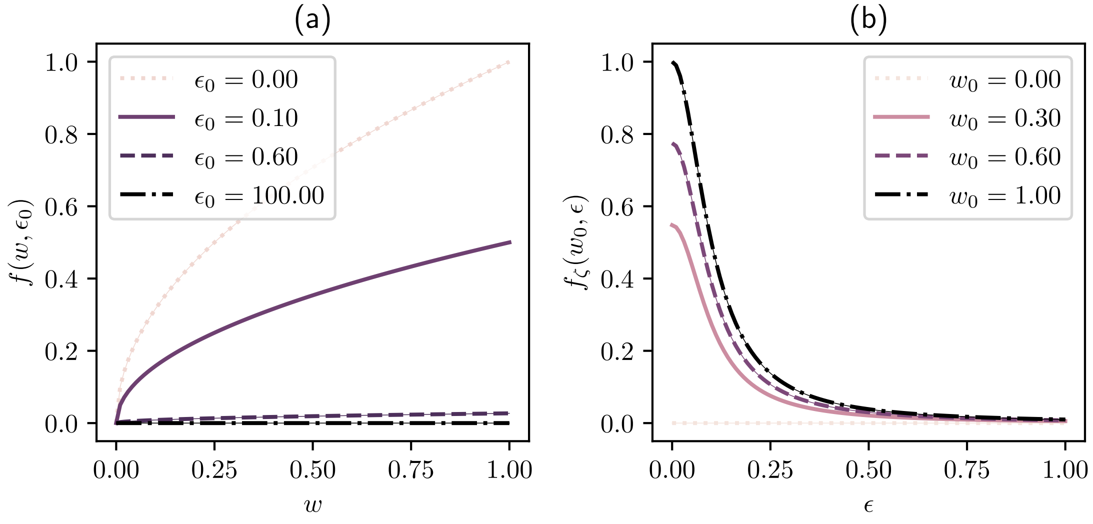

Figure S11: Sections of the surface defined by $\Gamma_\zeta(w, \epsilon)$: (a) Γ at fixed energy penalty ϵ_0 and (b) Γ at fixed metastability window width w_0. The examples are computed at $\zeta = 0.1$.

The parameter ζ, measured in the same unit as the energy penalty ϵ (here eV/site), sets the relative weight of the two arguments. Its effect is evident by the limiting behaviours. For small ζ, $\lim_{\zeta\to 0} \Gamma \approx \zeta^2 \frac{\sqrt{w}}{\epsilon^2}$, a large energy penalty lowers the score, within the upper-limit given by the window size $\sqrt{w}$. On the other hand, for large ζ, $\lim_{\zeta\to\infty} \Gamma \approx \sqrt{w}$ the largest window is always selected regardless of the energy price. The parameters ζ must be tuned according to the dataset considered in order to achieve the right sensitivity, i.e. a trade-off between large but costly windows and low energy solutions defined on a narrow concentration range.

1. Weight Selection for Optimal Host Matrix

Using the ranking function $\Gamma_\zeta(w, \epsilon)$ defined in the main text, the metastability windows in the matrices in Figures S1 to S4 can be ranked, yielding the optimal prototype for each pair. Left plots in Fig. S12 show all metastability windows from all entries the matrices in Figures S1 to S4 in the w, ϵ space. Each point in Figures S12a to S12c is colored according to its score for increasing values of ζ in $\Gamma_\zeta(w, \epsilon)$. Colored lines report isolines of $f_\zeta(w, \epsilon)$. The two limiting behaviour, $\zeta \to 0$ and $\zeta \to \infty$, mention in the previous section are clearly

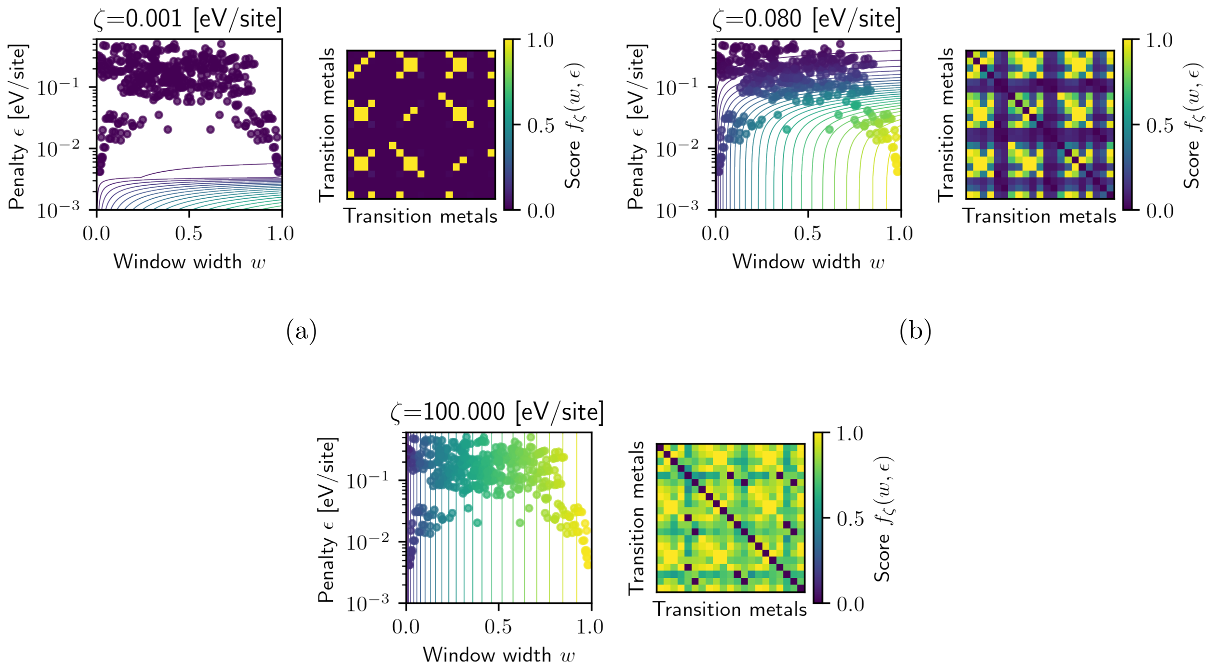

(a) (b)

(c)

Figure S12: Behaviour of the ranking function within the metastability windows dataset for (a) $\zeta = 1 \times 10^{-3}$ eV/site, (b) $\zeta = 8 \times 10^{-2}$ eV/site, and (c) $\zeta = 1 \times 10^{2}$ eV/site. Left plots, scatter plot of metastability windows in width w and energy cost ϵ space. Marker colors are assigned by $\Gamma_\zeta(w, \epsilon)$. Colored lines are isolines of $\Gamma_\zeta(w, \epsilon)$. Right plots show the value of Γ for the highest-scoring prototype for each TM pair.

visible. For small $\zeta = 1 \times 10^{-3}$ eV/site, Fig. S12a, all windows with non-zero energy penalty are assigned a low score, dark element on the right heat map, while only TM pairs with $(w, \epsilon) = (1, 0)$ show the non-vanishing scores, yellow entries on the right heat map. On the other hand, for $\zeta = 1 \times 10^{2}$ eV/site, Fig. S12c, the isolines of Γ dived the data points in vertical stripes. Thus, only the window width determines the score, resulting in the largest windows being picked as optimal prototypes, as shown in the right plots. The value adopted in this work is $\zeta = 0.080$ eV/site, which achieves a balance between low energy cost and wide metastability window, as shown in Fig. S12b. Somewhat surprisingly, the ranking is quite robust against the value of ζ: going from $\zeta = 1 \times 10^{-3}$ eV/site to $\zeta = 1 \times 10^{2}$ eV/site only 12% of optimal prototypes change.

VI. OPTIMAL PROTOTYPE MATRIX DETAILS

Figures S13 to S15 report the Pettifor maps in Fig. 4 in the main text without rotation and example marks.

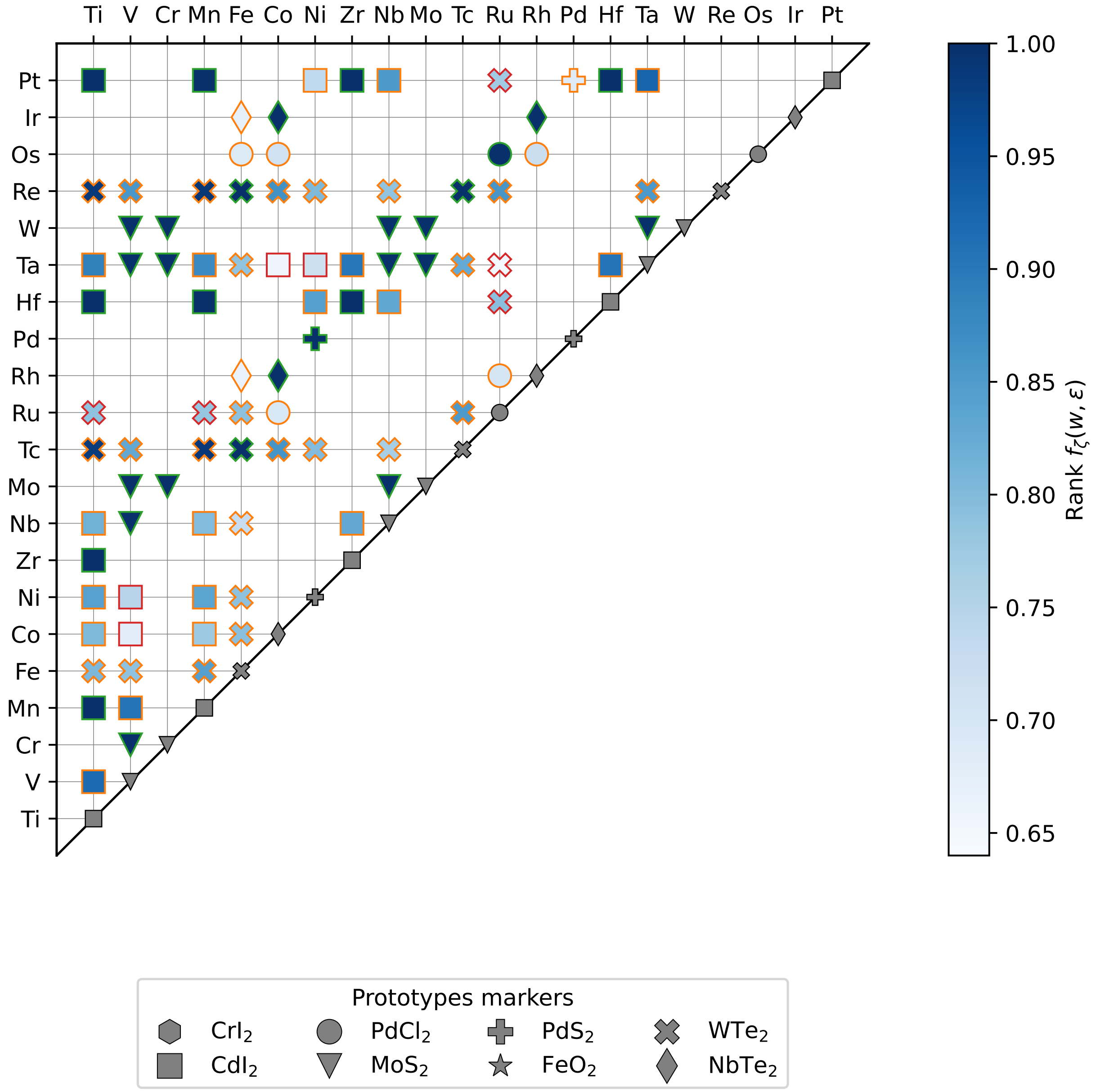

Figure S13: Pettifor map for optimal prototype for sulphides pairs.

Fig. S16 reports the metastability metric of the optimal prototype at fixed chalcogenides and Fig. S17 at fixed metal.

Fig. S18 reports the frequency of the optimal prototype at fixed chalcogenides and metals.

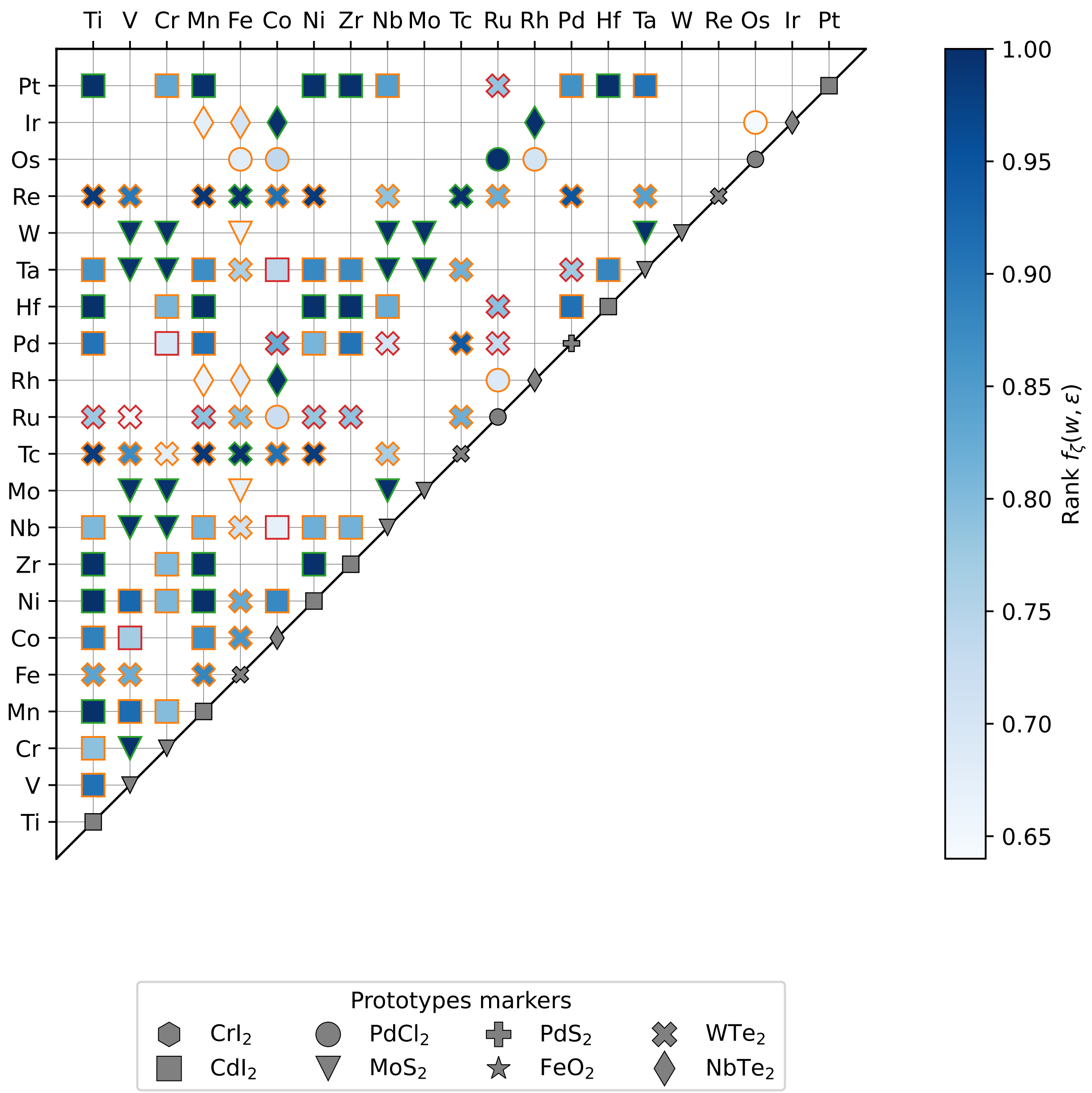

Figure S14: Pettifor map for optimal prototype for selenides.

Note that going from S to Se the share of pairs without an optimal prototype decreases, showing in a quantitative way the discussion in the main text.

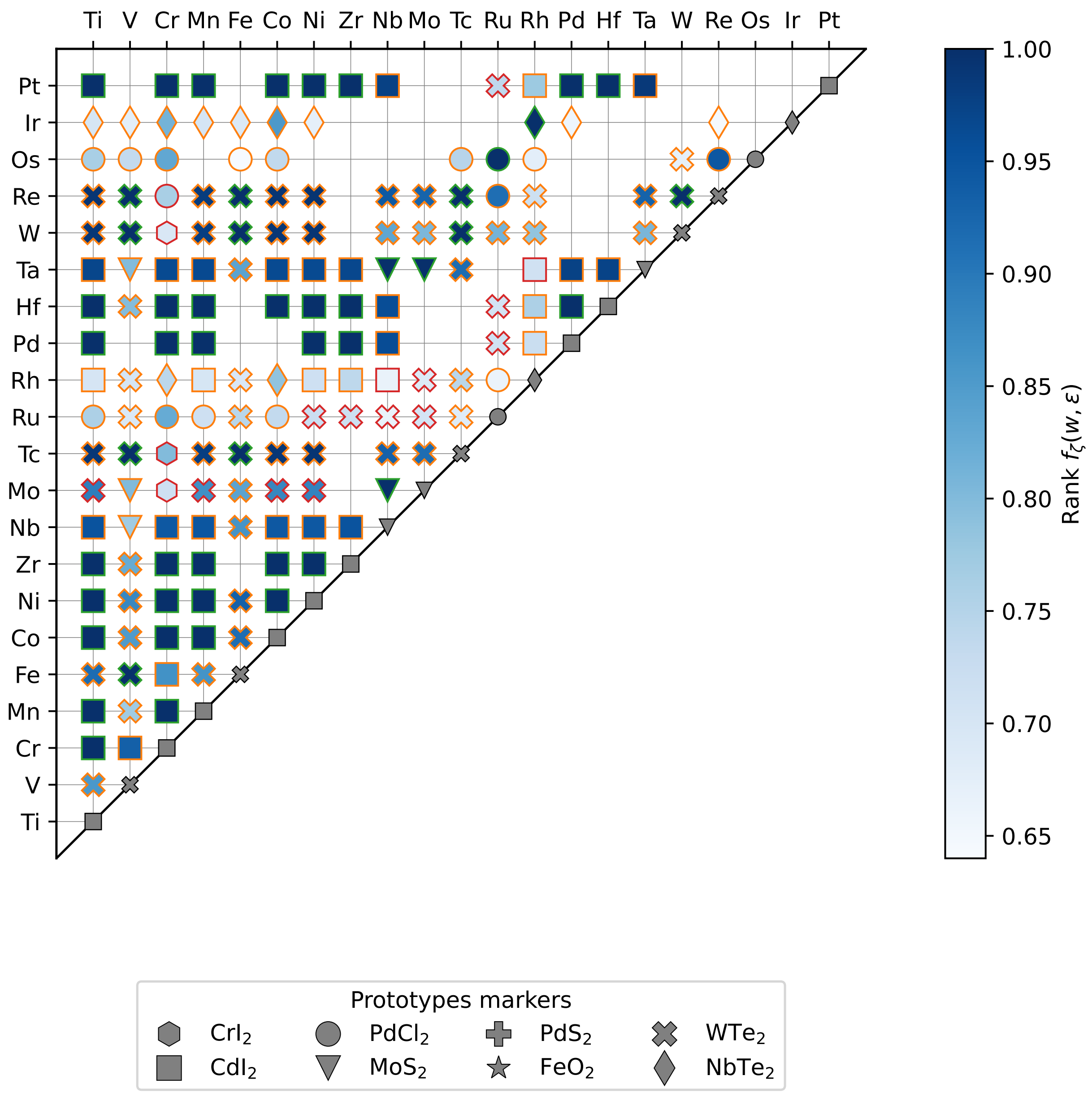

Figure S15: Pettifor map for optimal prototype for tellurides.

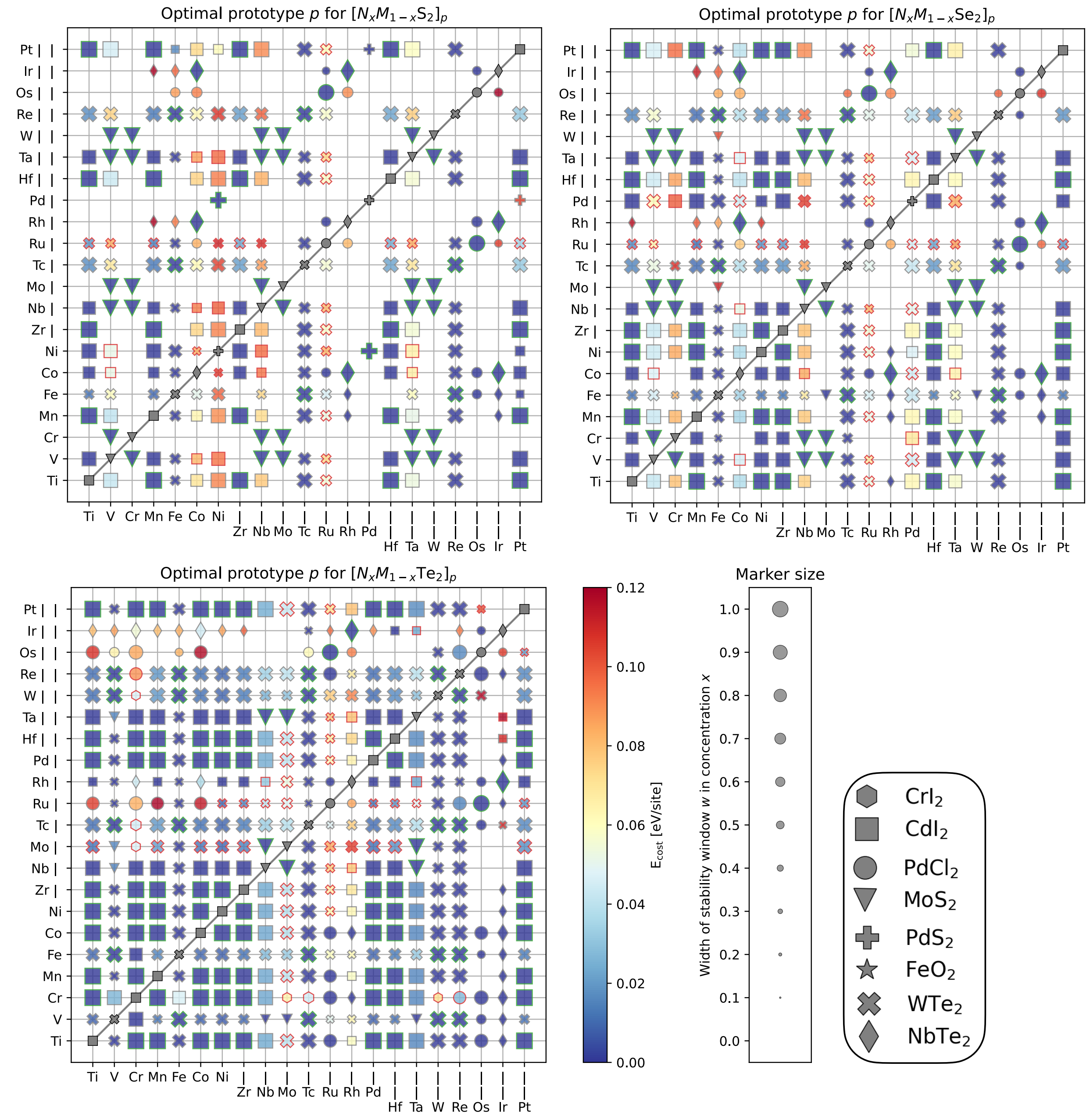

Figure S16: Optimal metastability metric at fixed chalcogen.

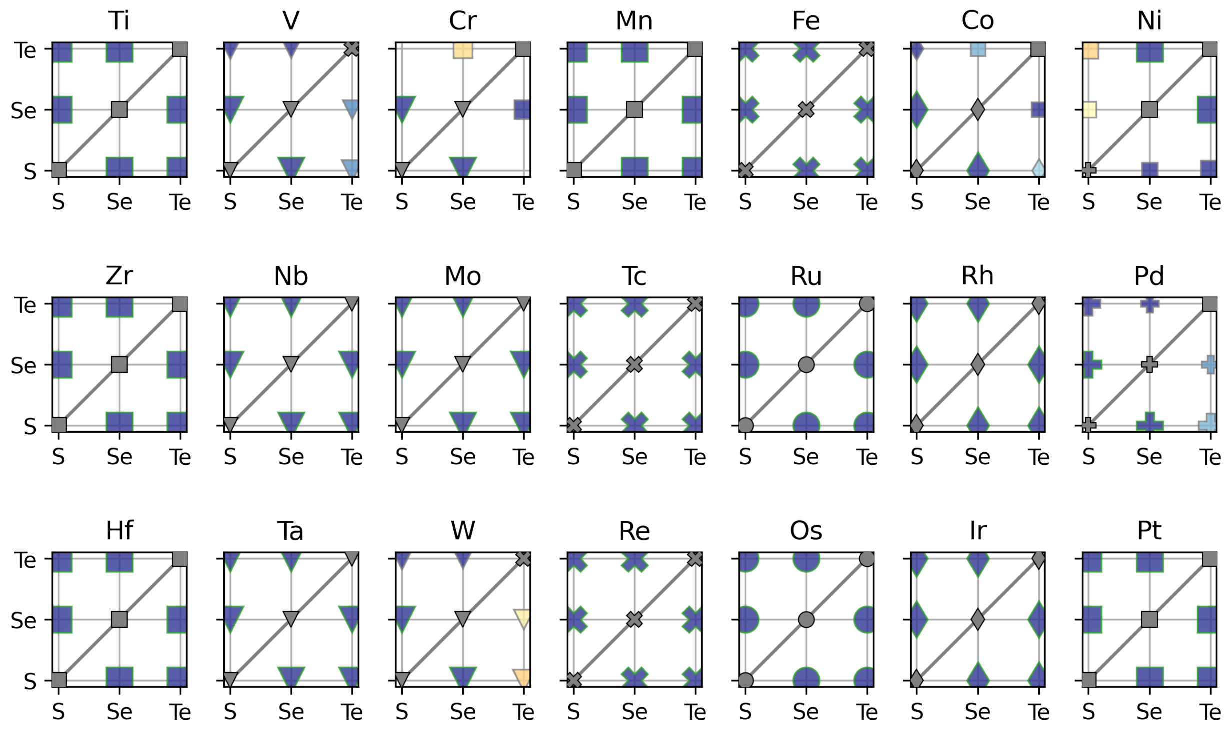

Figure S17: Optimal metastability metric at fixed metal.

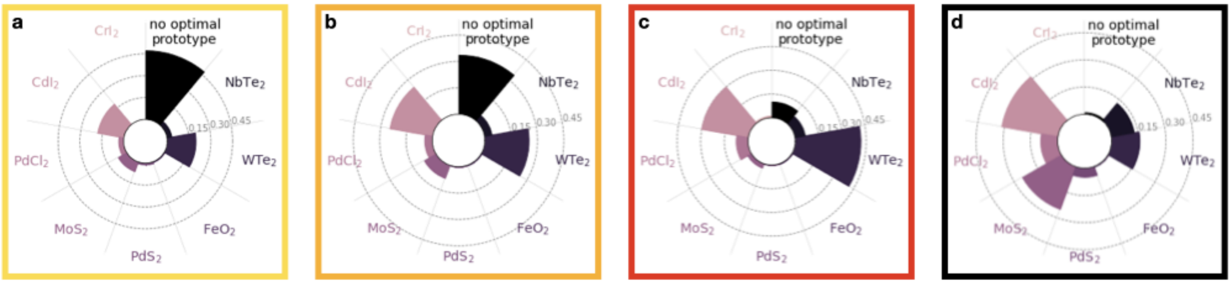

Figure S18: Frequency of optimal prototypes TMDs binaries alloys on the metal site in (a) sulphides, (b) selenides, (c) tellurides and (d) on the chalcogen site.

VII. CONFIGURATIONS SAMPLING DETAILS

The set of geometrically distinct configurations is generated using CASM [8–10]. The number of configurations at each concentration relates to the sampling density of the configurational space. In principle, in an infinite system there are infinite configurations at each concentration. As we are limited to finite-size supercells by computational power, only a handful of these infinite configurations are sampled with DFT calculations. Certain concentrations can be sampled better for a given supercell size, i.e. the number of repetitions of the crystal unit cell. This concept is illustrated in the toy system in Fig. S19 for a simple square lattice, a 2x2 supercell can host two distinct configurations at x=0.5, only one at x=0.25, one at x=0.75 and none at x=0.3. The spread at each concentration relates to the different energies associated with different atomic arrangements at the same concentration, e.g. alternated atomic rows (striped pattern) might have lower energy than a 2x2 arrangement (checkerboard pattern), as in Fig. S19. For more details about the configuration enumeration and how to identify geometrically equivalent arrangements see CASM [8–10]

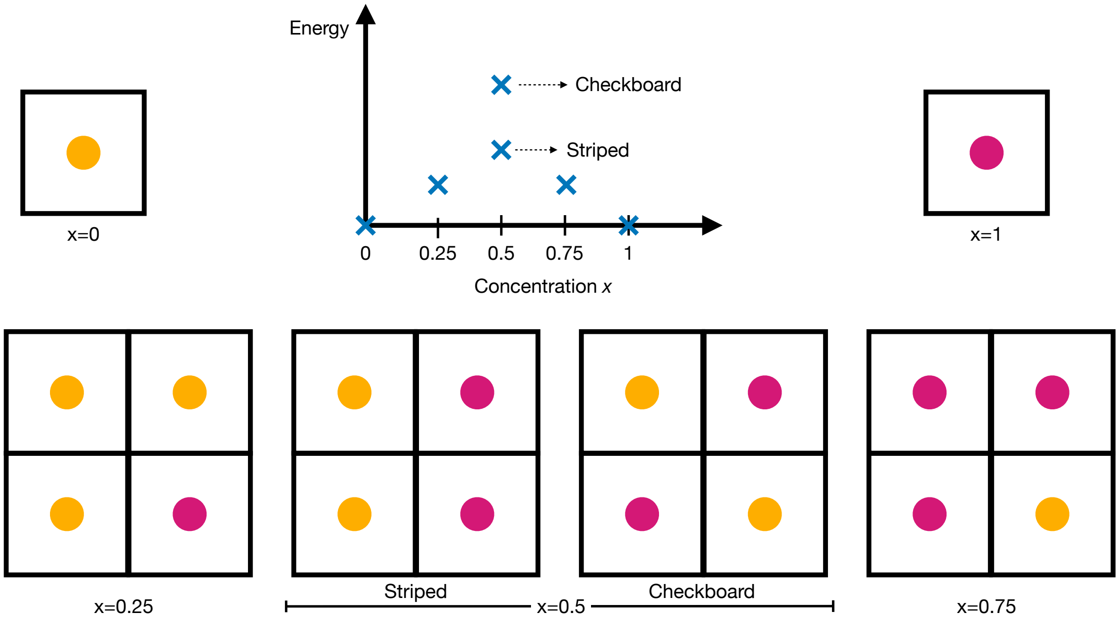

Figure S19: Example of geometrically distinct configurations for a simple square lattice up to 2x2 supercells.

Table SVI report the details of the DFT configurations computed for each alloy system presented in Section V of the main text. Additional details on (Mo:Ti)S_2 and (To:Ti)S_2 can

be found in Ref. [11, 12], respectively.

System	#DFT
(Mo:Ti) $S_2\|_{MoS_2}$	57
(Mo:Ti) $S_2\|_{CdI_2}$	113
(Ti:Ta) $S_2\|_{MoS_2}$	172
(Ti:Ta) $S_2\|_{CdI_2}$	396
W(Se:Te)$_2$ $\|_{MoS_2}$	1120
W(Se:Te)$_2$ $\|_{WTe_2}$	3507
(Mo:Ti) $Te_2\|_{MoS_2}$	174
(Mo:Ti) $Te_2\|_{CdI_2}$	215
(Mo:Ti) $Te_2\|_{WTe2_2}$	122

Table SVI: The first column reports the name of the system considered, the second column, #DFT, reports the number of *ab-initio* calculations comprising the training set.

A. (Mo:Ti)Te_2 details

A complication arises in the sampling of octahedrally coordinated structures derived from p-WTe_2 and p-CdI_2, respectively, because symmetry-breaking distortions can lead seamlessly from p-CdI_2 to p-WTe_2. Hence, it is not strictly possible to assign configurations after relaxation to either of the two prototypes. However, it is possible to define order parameters for each of the prototypes to measure the similarity of a given configuration with either. Different approaches are discussed in the literature to define similarity measures. For instance, Zimmermann and Jain developed a framework of local coordination measures [13], and the group of Van der Ven developed an elegant formalism taking into account lattice distortions as well as atom displacements [14]. Both are conveniently utilised, because the former is implemented in the pymatgen library [15], while the later is part of CASM [8–10], which we have used to generate symmetry in-equivalent orderings. The octahedral coordination measure implemented in pymatgen, however, did not yield satisfactory results in our case, and algorithm in CASM produced errors that we could not resolve.

We have, therefore, implemented a quick and simple measure similar to the popular SOAP descriptors [16, 17]. We define a similarity measure between a reference structure r and a structure s

$$f^r(s) = f^r_{\mathrm{bd}}(s) \cdot f^r_{\mathrm{ba}}(s)$$

as the product of a bond distribution measure f_{bd} and an bond angle measure f_{ba}. Each of these measures is defined as the normalised inner product of bond distribution and bond angle histograms, respectively. We define a feature histogram $h_j(x)$ for feature j as

$$h_j(x) = \{x\}_j * g_\sigma,$$

where each feature x_i in the set $\{x\}_j$ generates a delta function $\delta_{x_i,x}$ that is broadened by convolution with a Gaussian kernel g_σ with standard deviation σ. The feature sets consist of (i) the bond distances between TMs and nearest-neighbour sites, normalised by the average bond distance around a TM sites, and (ii) all bond angles X-TM-X around TM sites with X denoting next nearest neighbours.

With the inner product $\langle u, w\rangle = \int_0^\infty uw\,dx$ over histograms u and w, the similarity between structures s and r with respect to a feature j is defined as

$$f^r_j(s) = \frac{\langle h^s_j, h^r_j\rangle}{\sqrt{\langle h^s_j, h^s_j\rangle \cdot \langle h^r_j, h^r_j\rangle}}$$

Fig. S20a reports the similarity between WTe_2 and CdI_2 prototypes for all configuration initialised in these two geometries in Fig. 6b in the main text. Fig. S20b reports the similarity between MoS_2 and CdI_2 prototypes for all configuration initialised in the prismatic MoS_2 geometries in Fig. 6b in the main text. The color of each point reports the concentration along the tie line.

Configuration initialised in the CdI_2 prototype (crosses in Fig. S20a) preserve this geometry, i.e. they cluster on the right side with few transforming to WTe_2. Most configurations initialised to WTe_2 transform to CdI_2 to low concentration: see the cluster of red circles in the bottom right side in Fig. S20a, corresponding to high similarity to the CdI_2 prototype at low Mo concentration. As the Mo concentration increases, most configurations preserve the original WTe_2 character, resulting in the circles gradually moving to the upper left corner (similarity to $WTe_2 \approx 1$) at high Mo content (blue color) in Fig. S20a.

On the other hand, configuration initialised in the prismatic MoS_2 prototype cannot trivially transform to the octahedral environment, hence all configurations in Fig. S20b

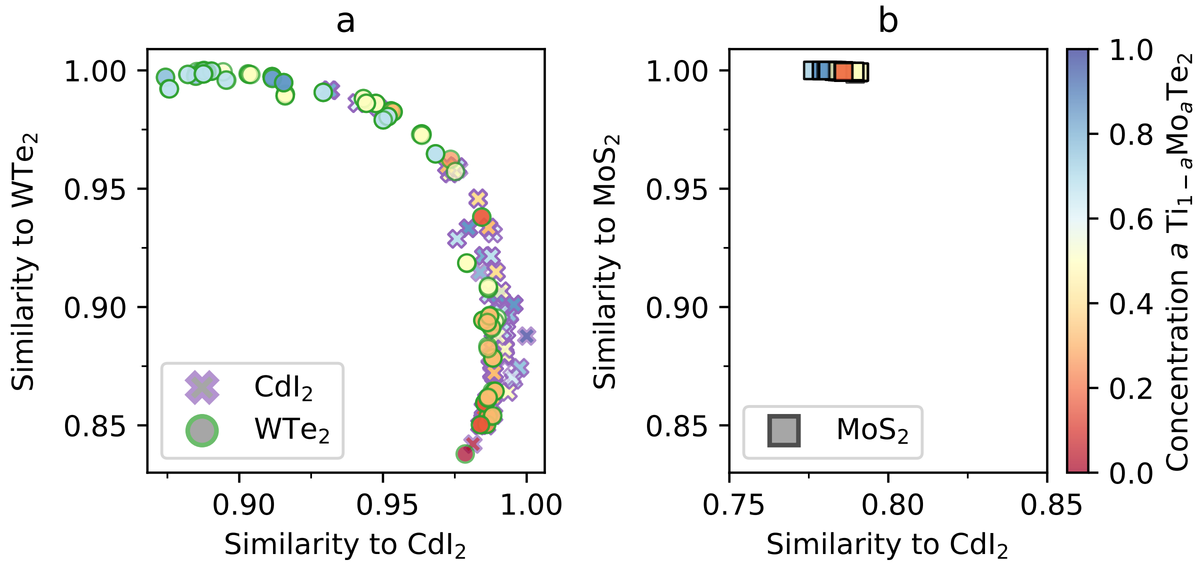

Figure S20: (a) Similarity between WTe2 and CdI2 prototypes for structures in the "octahedral-like" prototype, i.e. the union of structure initialised as WTe2 and CdI2. (b) Similarity between MoS2 and CdI2 prototypes for structures as MoS2.

cluster around height 1 (high similarity with MoS_2) and fixed similarity to $CdI_2 \approx 0.75$ regardless of the concentration.

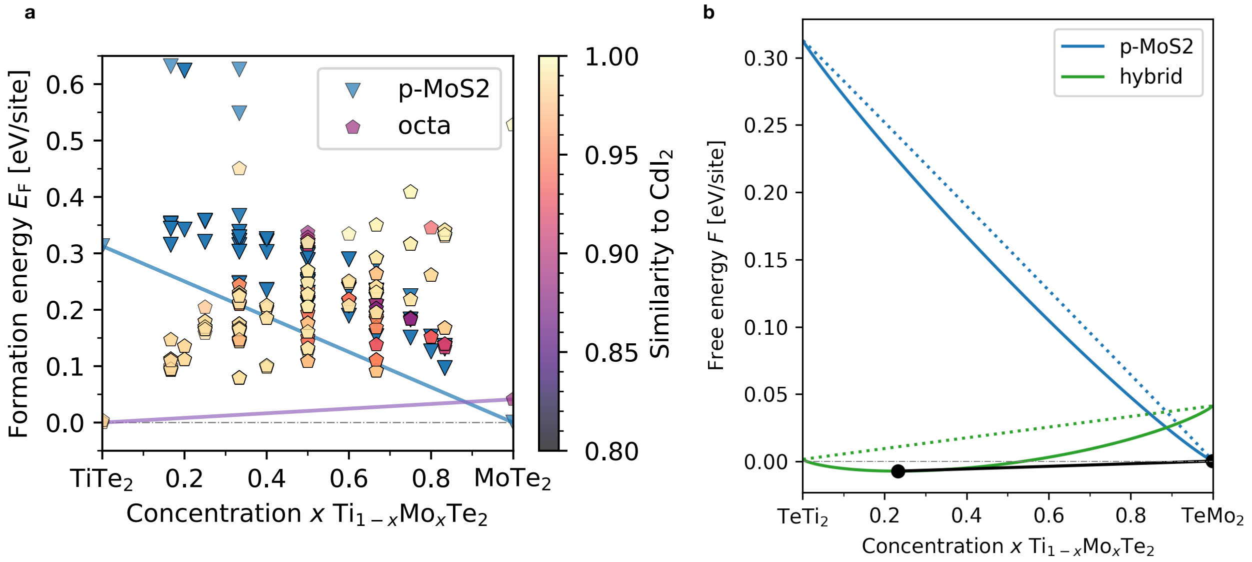

Figure S21: (a) Convex hull with hybrid "octahedral-like" prototype. (b) Finite temperature phase stability

Figure 21 (a) shows the resulting convex hull where the octahedrally coordinated configurations are designated by hexagons and marker color reflecting the similarity with p-CdI_2.

Figure 21 (b) shows an estimate of the free energy curves in prismatic and octahedral coordination. These have been generated using a Boltzmann-weighting strategy to obtain the energy U from the set of DFT computed energies and assuming ideal solid-solution behaviour for entropic contributions to the free energy. The Boltzmann-weighting of the energy is performed by:

$$U(x) = \frac{\sum E_i \cdot \exp(-\beta(E_i - x_i \cdot \mu(x)))}{\sum \exp(-\beta(E_i - x_i \cdot \mu(x)))},$$

which is akin to a semi-grand canonical average. But we stress that this is not a thermodynamically precise definition, because we sample only a small portion of the configurational space. In practice, we lack many configurations close to the hull and therefore risk to give too much weight to configurations with larger distances from the hull. In our approximate scheme we overcome this by treating β as a fitting parameter larger than $1/kT$ to reduce the over-weighting of high energy structures stemming from the constrained configuration space. Finally, $\mu(x)$ is formally obtained by inverting

$$\beta \cdot x(\mu) = \partial_\mu \ln \left(\sum \exp(-\beta(E_i - x_i \cdot \mu)) \right).$$

In practice, we compute $U(\mu)$ and $x(\mu)$ and then parametrically plot $U(\mu)$ over $x(\mu)$.

The free energy, is then obtained in the usual way from

$$F = U - T \cdot S_{\mathrm{id}} \text{with} \quad S_{\mathrm{id}} = -k_{\mathrm{b}} \left(x \ln(x) + (1-x) \ln(1-x) \right).$$

B. $W(Se{:}Te)_2$ details

We used the same Boltzmann-weighting strategy to estimate the bandgab as a function of composition in the $W(Se{:}Te)_2$ system. Using all DFT computed bandgaps shown in Figure 22 as the sets $\{E_b\}_j$, the bandgap as a function of composition is computed according to

$$E_b(x) = \frac{\sum E_{b,i} \cdot \exp(-\beta(E_i - x_i \cdot \mu(x)))}{\sum \exp(-\beta(E_i - x_i \cdot \mu(x)))}$$

In accordance with the respective graph in the main text, low energy configurations in p-WTe_2 plotted in Figure 22 (b) show negligible bandgaps, while low energy configurations

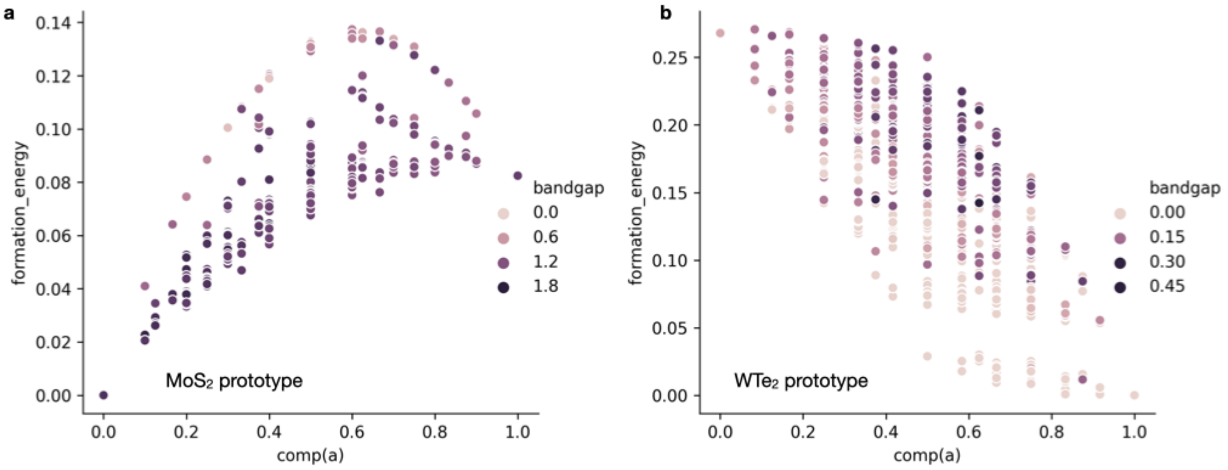

Figure S22: Bandgap for each computed structure in the (a) MoS_2 prototype and (b) WTe_2 prototype.

in p-MoS_2 have large bandgaps as shown in plot (a). This is reflected in the Boltzmann-weighted averages shown in the main text.

---

[1] N. Mounet, M. Gibertini, P. Schwaller, D. Campi, A. Merkys, A. Marrazzo, T. Sohier, I. E. Castelli, A. Cepellotti, G. Pizzi, and N. Marzari, Two-dimensional materials from high-throughput computational exfoliation of experimentally known compounds, Nature Nanotechnology **13**, 246 (2018).

[2] G. Bergerhoff, I. D. Brown, F. Allen, and others, Crystallographic databases, International Union of Crystallography, Chester **360**, 77 (1987).

[3] A. Vaitkus, A. Merkys, and S. Gražulis, Validation of the Crystallography Open Database using the Crystallographic Information Framework, Journal of Applied Crystallography **54**, 661 (2021).

[4] A. Togo and I. Tanaka, First principles phonon calculations in materials science, Scripta Materialia **108**, 1 (2015).

[5] Y. Chen, J. Xi, D. O. Dumcenco, Z. Liu, K. Suenaga, D. Wang, Z. Shuai, Y. S. Huang, and L. Xie, Tunable band gap photoluminescence from atomically thin transition-metal dichalcogenide alloys, ACS Nano **7**, 4610 (2013).

[6] X. Xia, S. M. Loh, J. Viner, N. C. Teutsch, A. J. Graham, V. Kandyba, A. Barinov, A. M.

Sanchez, D. C. Smith, N. D. Hine, and N. R. Wilson, Atomic and electronic structure of two-dimensional Mo(1-x)WxS2 alloys, JPhys Materials **4**, 10.1088/2515-7639/abdc6e (2021).

[7] B. Pattengale, Y. Huang, X. Yan, S. Yang, S. Younan, W. Hu, Z. Li, S. Lee, X. Pan, J. Gu, and J. Huang, Dynamic evolution and reversibility of single-atom Ni(II) active site in 1T-MoS2 electrocatalysts for hydrogen evolution, Nature Communications **11**, 4114 (2020).

[8] A. Van der Ven, J. C. Thomas, Q. Xu, and J. Bhattacharya, Linking the electronic structure of solids to their thermodynamic and kinetic properties, Mathematics and Computers in Simulation **80**, 1393 (2010).

[9] B. Puchala and A. Van Der Ven, Thermodynamics of the Zr-O system from first-principles calculations, Physical Review B - Condensed Matter and Materials Physics **88**, 1 (2013).

[10] J. C. Thomas and A. V. D. Ven, Finite-temperature properties of strongly anharmonic and mechanically unstable crystal phases from first principles, Physical Review B - Condensed Matter and Materials Physics **88**, 1 (2013).

[11] A. Silva, T. Polcar, and D. Kramer, Phase behaviour of (Ti:Mo) S2 binary alloys arising from electron-lattice coupling, Computational Materials Science **186**, 110044 (2021).

[12] A. Silva, J. Cao, T. Polcar, and D. Kramer, Pettifor maps of complex ternary two-dimensional transition metal sulfides, npj Computational Materials **8**, 10.1038/s41524-022-00868-7 (2022).

[13] N. E. Zimmermann and A. Jain, Local structure order parameters and site fingerprints for quantification of coordination environment and crystal structure similarity, RSC Advances **10**, 6063 (2020).

[14] J. C. Thomas, A. R. Natarajan, and A. Van der Ven, Comparing crystal structures with symmetry and geometry, npj Computational Materials **7**, 10.1038/s41524-021-00627-0 (2021).

[15] S. P. Ong, W. D. Richards, A. Jain, G. Hautier, M. Kocher, S. Cholia, D. Gunter, V. L. Chevrier, K. A. Persson, and G. Ceder, Python Materials Genomics (pymatgen): A robust, open-source python library for materials analysis, Computational Materials Science **68**, 314 (2013).

[16] A. P. Bartók, R. Kondor, and G. Csányi, On representing chemical environments, Physical Review B - Condensed Matter and Materials Physics **87**, 10.1103/PhysRevB.87.184115 (2013).

[17] S. De, A. P. Bartók, G. Csányi, and M. Ceriotti, Comparing molecules and solids across structural and alchemical space, Physical Chemistry Chemical Physics **18**, 13754 (2016).
